# Direct electrochemistry as a mechanistic tool for studying engineered myoglobins: implications on carbene transferase activity

**DOI:** 10.1039/d6dt00266h

**Published:** 2026-06-16

**Authors:** Evelina Venckute, Amanda G. Jarvis, Patricia Rodríguez-Maciá

**Affiliations:** a EaStCHEM School of Chemistry, University of Edinburgh Joseph Black Building David Brewster Road Edinburgh EH9 3FJ UK Amanda.Jarvis@ed.ac.uk; b School of Chemistry and Leicester Institute of Structural and Chemical Biology, University of Leicester Leicester UK prm28@leicester.ac.uk

## Abstract

Direct electrochemistry tools offer exclusive insight into the redox properties of metalloenzymes. However, they have not found widespread use within the artificial enzyme community, despite their potential to help inform catalyst design. Herein, we describe the use of a simple and robust method for non-covalent immobilisation of a library of myoglobin-based (Mb) engineered and cofactor-substituted metalloenzymes onto carbon electrodes. This allowed the determination of the reduction potentials (*E*_1/2_) spanning M(iii)/M(ii) redox states for Fe and Mn Mb systems. In Fe-containing systems, more positive *E*_1/2_ values matched higher carbene transferase activity under the tested conditions. For example, when *E*_1/2_ = –10.6 mV (*vs.* standard hydrogen potential, SHE), a total turnover number (TTN) of 230 was observed, whereas when *E*_1/2_ = –72.9 mV (*vs.* SHE), TTN = 16 in styrene cyclopropanation reactions. Additionally, we investigated the effect of active-site mutations on *E*_1/2_ and the enantioselectivity of the enzymes. We found that a single mutation of residue 64 from histidine to glycine (H64G) reversed the enantiopreference of the enzyme. This proof-of-concept study highlights the use of direct electrochemistry as a fast and efficient mechanistic tool for characterising and informing the engineering of ArMs for applications in sustainable catalysis and electrochemical transformations.

## Abstract 2

Direct electrochemistry tools offer exclusive insight into the redox properties of metalloenzymes.

## Graphical abstract

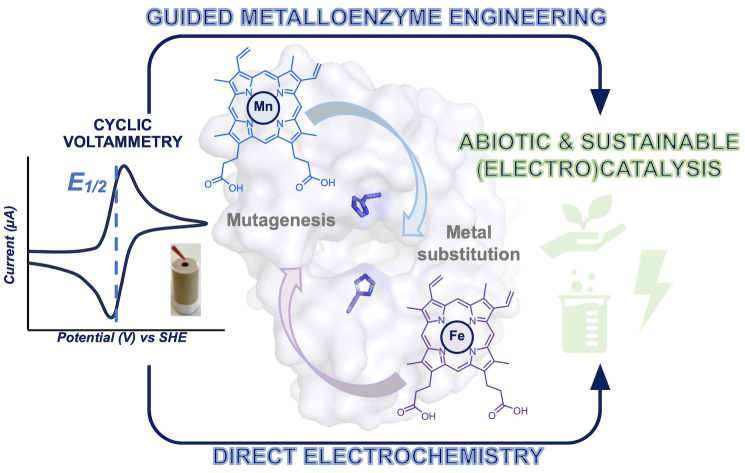

## Introduction

Myoglobin (Mb) is a 18 kDa monomeric globular hemoprotein with a native function in O_2_ storage. Due to its malleable architecture it has been used as a model scaffold in various hemoprotein function studies as well as developed into a diverse array of ArM catalysts.^1,2^ Recently designer Mb ArMs have achieved non-native and abiotic activities using active site engineering strategies, such as cofactor substitution with different metal porphyrin complexes.^3–6^ Electrochemical tools can be used to screen and tune the redox properties of metalloenzymes, including ArMs.^7^ Despite such measurements remaining scarce in current ArM research, they demonstrate great potential in selectively guiding further engineering of ArM libraries for target applications in redox transformations, such as biosensing and activation of small molecules.

**Fig. 1:**
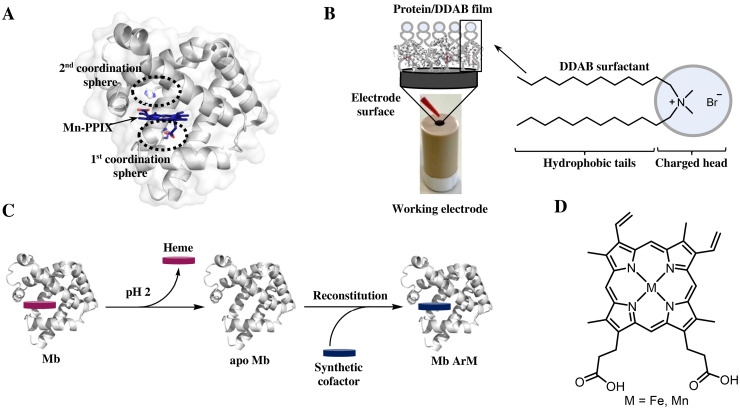
(A) Model of Mn-substituted MbQ variant protein scaffold (generated using PyMOL and MbQ scaffold from Fe-MbQ X-ray structure, PDB: 5OJA). MbQ – variant of sperm whale Mb with the following mutations: T39I, R45D, F46L and I107F. (B) Protein immobilisation on the electrode using DDAB surfactant. (C) Procedure for preparation of Mb ArMs. (D) Protoporphyrin IX complexes used in this work.

Direct electrochemistry methods, by definition, permit access to non-mediated direct electrochemical measurements of metalloprotein redox properties and catalytic activity.^8^ To date, many direct electrochemistry studies of wild-type (WT) Mb have relied on the use of modified electrodes and covalent protein-immobilisation strategies to address the non-electron transfer nature of globins.^8–10^ However, these methods are often very specialised and require additional synthetic modification procedures for functionalization of both proteins and electrodes. The use of surfactants, such as didodecyldimethylammonium bromide (DDAB), was pioneered in the 1990s and allowed proteins that are not inherently involved in electron transfer to be studied by direct electrochemical methods.^11^ DDAB forms bilayer films on electrode surfaces, creating a lipid-like environment that can entrap proteins, stabilizing them and bringing them into electrical contact with the electrode.^12^ Despite the use of these techniques to characterise natural metalloenzymes, their use in ArM characterisation and design remains scarce, with previous work mostly focusing on the use of indirect electrochemical methods.^13,14^ For example, Fasan *et al.* reported a redesigned Fe porphyrin cofactor, the Fe-2,4-diacetyl deuteroporphyrin IX, for a myoglobin-based ArM for the stereoselective cyclopropanation of electron-deficient olefins with diazoesters, using indirect electrochemistry methods for their characterisation.^15^ Whilst these methods are valuable, they limit the study of their electron transfer and electrocatalytic properties. In this work, we took inspiration from the work done by Rusling, Li and others on the use of DDAB^11^ and other direct electrochemistry methods^16,17^ for characterisation of metalloproteins, to develop a robust and straightforward method for non-covalent adsorption of engineered myoglobins on a pyrolytic graphite electrode (PGE) using DDAB surfactant to determine their redox properties under a range of conditions (*i.e.*, different pH values) using direct electrochemistry. This approach enabled examination of 1^st^- and 2^nd^-coordination sphere Mb mutants bearing either Fe(iii) or Mn(iii) redox cofactors (Fig. 1A) under identical conditions, allowing direct comparison of their redox properties and correlation with catalytic performance. Surfactant-embedded proteins were characterised using cyclic voltammetry (CV) to probe their redox potentials (*E*_1/2_) and electrochemical behaviour (Fig. 1B). Importantly, our study shows that direct electrochemistry is a valuable tool for obtaining *E*_1/2_ values and allows correlations between Mb active-site architecture, *E*_1/2_ values, and catalytic activity to be drawn.

## Results and discussion

### Characterisation of Fe- and Mn-MbQ

The sperm whale MbQ variant containing four active site mutations (T39I, R45D, F46L and I107F), previously reported for its robustness and tolerance to synthetic modifications, was selected as a target scaffold for further biohybrid catalyst development and electrochemical screening.^18^ Heme-free MbQ (apo MbQ) was prepared using an acid/butanone extraction method and then reconstituted with either Fe-PPIX-Cl or Mn-PPIX-Cl (Fig. 1C and D).^19^ Biophysical characterisation of apo MbQ, Fe-PPIX bound MbQ (Fe-MbQ) and Mn-PPIX bound MbQ (Mn-MbQ) was performed using UV/visible and circular dichroism (CD) spectroscopy and native nano-electrospray ionisation mass spectrometry (native nanoESI-MS) methods. Together these methods confirmed removal of the heme, incorporation of synthetic cofactor and retention of the native-like fold in the MbQ matrix (Fig. S1–S14).

**Fig. 2:**
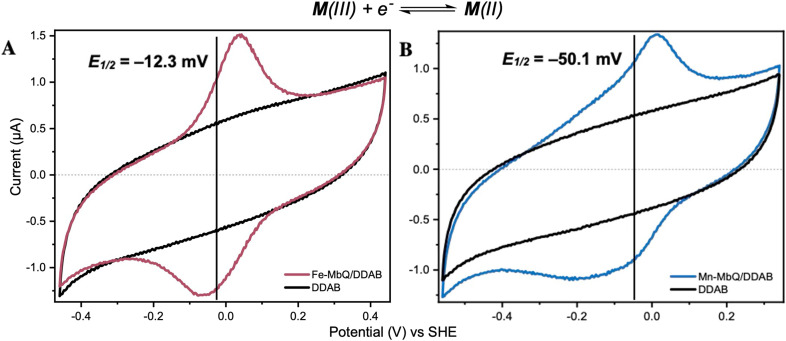
CVs of (A) Fe-MbQ and (B) Mn-MbQ adsorbed in DDAB films on PG electrodes. Measurements were performed under N_2_ atmosphere at 25 °C in buffer mix solution (pH 7) using 0.1 V s^−1^ scan rate. Dashed horizontal line marks zero current, and solid vertical lines represent the redox midpoint potentials (*E*_1/2_).

With the successfully reconstituted metalloproteins in hand we looked at their electrochemical characterisation. Slow electron-transfer kinetics between the redox centre and the electrode surface are commonly observed in metalloproteins where the redox centre is embedded within the protein cavity, as in Mb.^8^ Hence, adsorption of Mb on the PGE in the absence of film additives is inefficient and gives no apparent peaks in the measured CVs (data not shown). To overcome this, we re-visited the literature and prepared a range of Mb films with polymer and nanoparticle additives. Initial attempts with BP2000 particles, carboxymethyl cellulose and polymyxin b were unsuccessful despite evidence in the literature on their ability to aid protein adsorption on Au modified electrodes and PGEs.^20–22^ Therefore, our main efforts were directed towards the use of bilayer forming polymers with a two-component structure (Fig. 1B).^23^ As mentioned above, such polymer resins can greatly improve adsorption of proteins bearing mixed surface charge, as is the case of Mb, onto hydrophobic electrode surfaces to enable electrical contact (Fig. S15).^11,24,25^ Using a three-electrode electrochemical cell set-up under anaerobic conditions in pH 7 buffer containing 100 mM NaCl supporting electrolyte, screening of either Fe-MbQ or Mn-MbQ films led to successful detection of the M(iii)/M(ii) redox couples (Fig. 2A and B). Formal potentials (*E*°′), calculated as the midpoint potentials (*E*_1/2_) between the cathodic and anodic peaks, were determined at −12.3 mV and −50.1 mV *vs.* SHE, for Fe-MbQ and Mn-MbQ DDAB-films respectively. The CV of Fe-MbQ was rather symmetrical, whereas that of Mn-MbQ was non-symmetrical and displayed a broader reduction peak. Overlaid Mn-MbQ and protein-free Mn-PPIX-Cl CVs showed some overlap in the reductive peaks, while the oxidative peaks were clearly different (Fig. S16). Fitting of the Mn-MbQ (Fig. S17A) and protein-free Mn-PPIX-Cl (Fig. S17B) CVs using QSoas revealed two species for both systems, all with *n*-values lower than 1. The low potential component in both cases is very similar (*E* = –169 mV in Mn-MbQ *vs. E* = –176 mV in free Mn-PPIX-Cl cofactor), while the high potential component is quite different (*E* = –63 mV in the free cofactor sample, while *E* = –25 mV in the Mn-MbQ sample) (see Fig. S17, and Table S3). This suggests that the Mn-MbQ and protein-free Mn-PPIX-Cl systems exhibit more complex redox behaviour than simple one-electron transitions. One potential explanation of the observed redox behavior could be that free cofactor is being released due to instability of the protein on electrochemical reduction, or as a result of chemical steps (*i.e.* protonation, or ligand binding such as water), however, the fitting of the data is not suggestive of free cofactor. Previous electrochemical studies of electrode-confined Mn-PPIX reconstituted Mb and cytochrome c peroxidase displayed similar broadening of the redox peaks in their respective CVs.^24^ However, in these studies the unusual redox behavior was attributed to two redox-active forms in each Mn-PPIX bound protein scaffold. In our hands, Fe-MbQ and Mn-MbQ system displayed diffusionless electron-transfer behavior, *i.e.* the measured redox-active species were electrode-confined, at scan rates at least up to 0.5 V s^−1^, as observed from the linear relationship between the measured peak current densities and the corresponding scan rates (Fig. S18 and S19). This confirmed that the Fe-MnQ-DDAB and Mn-MbQ-DDAB films were strongly adsorbed on the electrode surface. Additionally, the peak-to-peak separation of approximately 60 mV suggest quasi-reversible behavior. Analogous measurements performed using protein-free cofactor adsorbed with DDAB (Table 1) demonstrated that encapsulation of Fe-PPIX and Mn-PPIX complexes within the MbQ scaffold shifted the *E*_1/2_ values to more positive values. Protein scaffold and solvent imposed changes in the electrostatics and polarity around the cofactor can significantly alter the free energy associated with the cofactor redox activity and tune the measured *E*_1/2_ values.^25,26^ More positive *E*_1/2_ values are often observed for hemoprotein complexes containing hydrophobic heme-binding cavities, including those comprised of globin and cytochrome folds, compared to constituent protein-free metal complex samples.^27–29^ This trend was maintained here – the protein binding was found to tune *E*_1/2_ positively by *ca.* 50–60 mV for Fe-PPIX and Mn-PPIX, in line with previous reports.^25,30^ Control CV measurements performed using DDAB surfactant and constituting components in the protein–cofactor assembly as well as DDAB films alone confirmed that under inert atmosphere and applied buffer and temperature conditions cofactor leaching was not observed.^30^ Overall we show that this method is suitable to probe both the redox properties of ArMs as well as the stability of protein cofactor assemblies as cofactor dissociation can be identified by changes to the redox potential. Importantly, our method required only small quantities of protein solution (2 μL of 0.5 mM solution) making it a valuable tool when only limited quantities of proteins are available.

**Table 1:** Direct electrochemistry analysis results^a^

Entry	Redox system	*E*_1/2_ (mV) *vs.* SHE
1	Fe-PPIX-Cl	−72.9
2	Fe-Mb^b^	−8
3	Fe-MbQ	−12.3
4	Mn-PPIX-Cl	−99.7
5	Mn-MbQ	−50.1

a Redox systems measured after immobilisation with DDAB surfactant on PG electrode under N_2_ atmosphere at 25 °C in buffer mix solution (pH 7) using 0.1 V s^−1^ scan rate.

b Mb from horse heart.

### Redox properties of Fe- and Mn-MbQ variants

To probe how 1^st^- and 2^nd^-coordination sphere residues tune the *E*_1/2_ values and redox properties, a set of Fe-PPIX-Cl and Mn-PPIX-Cl reconstituted MbQ variants targeting the distal and proximal histidine residues, His64 and His93 respectively, were prepared. His93 directly coordinates the Fe-PPIX in the WT Mb, and His64 is essential for substrate activation.^31,32^ The histidine residues were mutated to glycine, the simplest amino acid bearing no side chain, and aspartic acid, an oxygen-donor amino acid, to produce the variants: H64D, H64G and H93D (Table 2). CV analysis of these variants immobilised with DDAB yielded varied results among the two groups of proteins containing either Fe or Mn redox centres, but importantly, mutation of H93 allows cofactor insertion. In the Fe systems, the MbQ H64D mutant maintained its *E*_1/2_ value similar to that of MbQ, whereas both H64G and H93D mutations shifted *E*_1/2_ negatively by at least 17.8 mV (Table 2). In the Mn systems, both proximal and distal mutations had a more significant effect on the measured *E*_1/2_ values. Here, the proximal H93D mutation shifted the *E*_1/2_ value relative to the MbQ parent scaffold positively by 67.5 mV, while the distal H64D and H64G mutations shifted *E*_1/2_ positively by 48.5 and 57.4 mV, respectively. Hence, installation of a strong π-donor ligand in the H93 position resulted in a greater Mn(ii) centre stability, although a similar effect on the redox potential values could also be attained by changing the electrostatics within the distal cavity.

**Table 2:** Direct electrochemistry analysis of Fe-MbQ and Mn-MbQ variants measured immobilised with DDAB surfactant on PG electrode under N_2_ atmosphere at 25 °C in buffer mix solution (pH 7) using 0.1 V s^−1^ scan rate

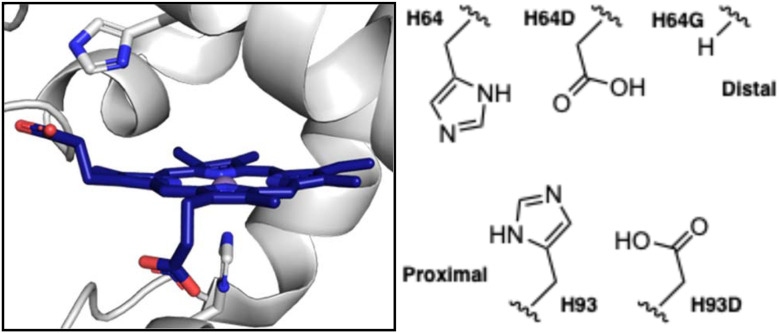			
Entry	Redox system	*E*_1/2_ (mV) *vs.* SHE	Δ*E*_1/2_ (mV) *vs.* Fe-MbQ (1–3) or Mn-MbQ (4–6)
1	Fe-MbQ H64D	−10.6	+1.7
2	Fe-MbQ H64G	−30.1	−17.8
3	Fe-MbQ H93D	−47.3	−35.0
4	Mn-MbQ H64D	+7.3	+57.4
5	Mn-MbQ H64G	−1.6	+48.5
6	Mn-MbQ H93D	+17.4	+67.5

**Fig. 3:**
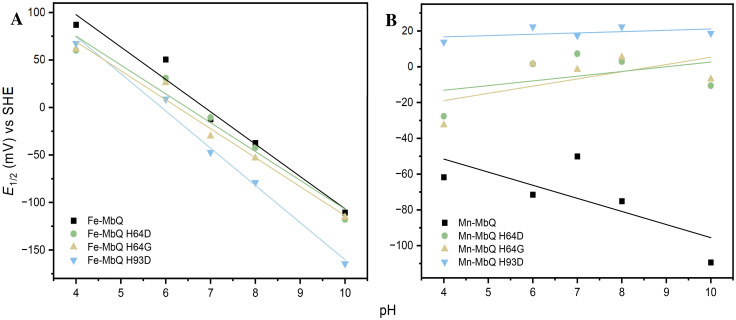
Impact of pH conditions on *E*_1/2_ values of (A) Fe-MbQ and (B) Mn-MbQ variants adsorbed in DDAB films on PG electrode surface. Measurements were performed under N2 atmosphere at 25 °C in varied pH buffer mix solutions using 0.1 V s^−1^ scan rate.

In many redox-active metalloenzymes, electron transfer is coupled to H^+^ transfer processes *via* the so-called proton-coupled electron transfer (PCET), and this can be probed by comparing changes in the *E*_1/2_ values over a pH range.^33,34^ PCET processes usually feature stoichiometric proton/electron ratios as evidenced by a Nernstian shift of 59.2 mV pH^−1^ unit. In our hands, the *E*_1/2_ values of all the Fe-MbQ variants across the pH range of 4–10 were measured at 33.8–42.2 mV per pH unit – *ca.* half of the Nernstian value (Fig. 3A). However, in all Mn-MbQ variants, across the pH 4–10 range, the *E*_1/2_ values varied only at *ca.* 7.3 mV per pH unit (Fig. 3B), suggesting that the electron transfer was not coupled to H^+^ transfer and that switching the identity of the metal centre changed the associated electron-transfer pathway. Interestingly, further screening performed using Mn-MbQ embedded within Nafion D-521 films, a perfluorinated two-compartment polymer widely used in proton exchange membrane fuel cells, electrolysers, sensors and electrocatalysis, improved the dependence of *E*_1/2_ on pH – it averaged at 28.5 mV per pH unit (Fig. S20). Hence, Nafion D-521 appears to facilitate PCET to and from the Mn centre. The sulfonic acid groups (–SO_3_H) on the polymer side chains dissociate in water, allowing proton transport. Thus, once the protein-Nafion D-521 films are hydrated, they are permeable to protons. This highlights that careful selection of conditions for direct electrochemistry screening can be used to rationally tune and optimize the electron-transfer properties of ArMs bearing non-native redox centres. Importantly, this can help improve their catalytic performance in target redox transformations, especially at non-ambient conditions. Overall, our results showed that mutating residues in the 1^st^- and 2^nd^-coordination spheres can tune the *E*_1/2_ and redox properties of the ArM systems. Although this holds true regardless of the metal identity at the active site, it seems to be less pronounced for Fe, and the mutants of Fe all display more negative potentials, while those for Mn display more positive potentials.

### Carbene transferase activity

Finally, the activity of the Fe-MbQ and Mn-MbQ variants in M(ii) centre orchestrated catalysis was evaluated using styrene cyclopropanation (Table 3). Under anaerobic conditions, the highest catalytic activity was observed for the Fe-MbQ H64D (determined as total turnover number (TTN) = 230) and Fe-MbQ variants (TTN = 121) both showing substantial increases in activity over the free cofactor (Fe-PPIX-Cl, TTN = 16). In comparison the Mn-MbQ variants were inactive (TTN <1, see Table S4) and no turnover was observed for the free Mn-PPIX-Cl complex. The relative activity of the two metal systems is in agreement with previous reports on the catalytic properties of Fe- and Mn-porphyrin Mbs (TONs <1 for Mn-bound WT Mb and singly mutated Mbs, Table S4).^3,4^

**Table 3:** Comparison of Fe catalyst activity in styrene cyclopropanation reaction^a^

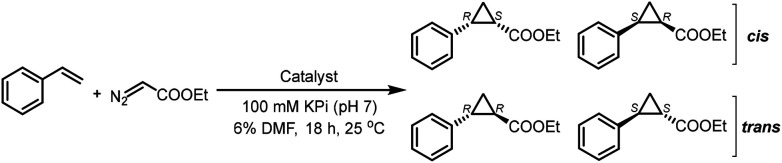				
Entry	Catalyst	TTN^b^	de_E_ c (%)	ee_E_ d (%)
1	Fe-PPIX-Cl	16 ± 4	75	−1
2	WT Mb^e^	60 ± 2	83	12
3	Fe-MbQ	121 ± 4	90	−68
4	Fe-MbQ H64D	221 ± 15	95	−79
5	Fe-MbQ H64G	96 ± 4	91	60
6	Fe-MbQ H93D	110 ± 4	81	−1

a General conditions: [catalyst] = 20 μM, [styrene] = 10 mM, [EDA] = 40 mM, [Na_2_S_2_O_4_] = 10 mM in 100 mM KPi buffer (pH 7) with 6% v/v DMF under N_2_ atmosphere at 25 °C for 18 h. Reactions setup using Fe-PPIX-Cl and Mn-PPIX-Cl contained 1% v/v DMSO.

b Total turnover number, *n* ≥ 3 replicates, TTN = ([*cis* products] + [*trans* products])/([catalyst]).

c Diastereomeric excess, *n* ≥ 3, calculated for the *trans* products where de = ([*trans*]-[*cis*])/([*trans*] + [*cis*]) × 100.

d Enantiomeric excess, *n* ≥ 3 replicates, calculated for (1*S*,2*S*)-cyclopropane in the *trans* products where ee_E_ = ([1*S*,2*S*]-[1*R*,2*R*])/([1*S*,2*S*] + [1*R*,2*R*]) × 100.

e Mb from sperm whale.

High trans diastereoselectivity was obtained across all the Fe Mbs. Intriguingly, we found that a single mutation at H64 switched the enantiopreference of the catalyst. The Fe-MbQ and Fe-MbQ H64D variants favoured the (1*R*,2*R*)-cyclopropane stereoisomer (ee_E_ = −68 and −79%, respectively). Meanwhile, the Fe-MbQ H64G mutant was selective for the (1*S*,2*S*)-cyclopropane stereoisomer in the *trans* products (ee_E_ = 60%). In P411 carbene transferases a single-mutation promoted switch in enantioselectively has been linked to altered substrate orientation within the distal cavity due to H-bonding interactions.^35^ It is possible that a similar interaction between the iron-carbene intermediate and the polar sidechains of the H64 and H64D residues could be invoked here to explain the observed switch in stereochemistry (Fig. S21). This explanation would also be consistent with the high enantioselectivities observed for the (1*S*,2*S*) product (ee up to 99%) when using the myoglobin double mutant (MbD = swMbH64V, V68A)^36^ where H64 is mutated to valine. However it is noted that further mutations to the swMb active site led to high *R*,*R* selectivities.^37^

The catalytic results for the Fe Mb variants were compared to the measured M(iii)/M(ii) reduction potentials. Within the Fe-MbQ system, variants exhibiting more positive *E*_1/2_ values displayed higher TTNs in styrene cyclopropanation (Table 3: Fe-MbQ H64D → TTN = 230, *E*_1/2_ = −10.6 mV *vs.* SHE; and Fe-MbQ → TTN = 121, *E*_1/2_ = −12.3 mV *vs.* SHE). In contrast, Fe-PPIX-Cl with the most negative *E*_1/2_ value showed the lowest activity (TTN = 16) compared to Fe-MbQ variants. These observations are consistent with recent reports demonstrating a positive correlation between the *E*_1/2_ values, determined using electron-transfer mediators, and carbene transferase activity in Mb-based ArMs incorporating synthetic porphynoid cofactors or noncanonical amino acid residues.^38,39^

## Conclusions

Reliable and reproducible protein-immobilisation methods for direct electrochemical characterisation make it an attractive tool for characterising ArMs, as it does not require additional electron-transfer partners alternatively used in indirect electrochemistry methodologies. Here, we used this technique as a proof-of-concept to enable the study of redox active Mb-based ArMs by direct electrochemistry, facilitating comparison of their redox properties and their catalytic performance under identical conditions. Our observation that more positive *E*_1/2_ values were associated with improved catalytic performance supports previous findings, suggesting that understanding the relationship between *E*_1/2_ and catalytic performance provides a useful and valuable parameter for guiding future ArM design. The ease and simplicity of the technique also make it readily extendable to other redox-active ArM systems enabling the investigation and direct comparison of the redox properties of WT, engineered, and artificial systems under non-ambient conditions while requiring only very small quantities of protein. Further studies on the electroenzymatic applications of immobilised and cofactor-substituted Mb ArMs are underway. Using a more extensive library of mutants, we aim to explore the potential of this method to guide the future development of ArMs for (electro)enzymatic methodologies with applications in sustainable catalytic transformations, including CO_2_ reduction and H_2_ evolution reactions.

## Methods

### Materials

All chemicals and biological reagents were purchased from commercial suppliers at the highest purity available and used without any further purification unless stated otherwise. Yeast extract was from VWR, 2xYT media was from MP Biomedicals, Kanamycin A sulfate from Cayman Chemical Company, LB agar (Miller), Tryptone plus, potassium dibasic phosphate, ammonium acetate, DMSO and sodium chloride from Fisher Scientific, Nafion-D521 dispersion (5% w/w in water and 1-propanol) and hemin from Alfa Aesar, MES, HEPES, TAPS and CHES from Melford, IPTG, imidazole from Fluorochem, horse heart Mb, Trizma base, sodium acetate, potassium monobasic phosphate, didodecyldimetrylammonium bromide (DDAB), DMF, styrene, ethyl diazoacetate and sodium dithionite from Sigma Aldrich, Mn-PPIX-Cl from Frontier Scientific.

### Preparation of Mb mutant variants

A pET29b(+) plasmid containing a gene encoding sperm whale MbQ with quadruple mutations (Thr39Ile, Arg45Asp, Phe46Leu, Ile107Phe) was kindly gifted by Professor Anthony Green (University of Manchester). Site-directed mutagenesis of MbQ active site residues was performed using the Q5 Site-Directed Mutagenesis Kit from New England BioLabs. Custom-designed oligonucleotide primers were synthesised by Integrated DNA Technologies. Oligonucleotide sequences (5′–3′): (1) H64G, *T*_m_ = 60 °C, forward GGTAACACCACCTTTTTTCAGATCTTCAGAAGCTTTC, reverse GTGTTAACTGCCCTAGGTG; (2) H64D, *T*_m_ = 60 °C, forward GTAACACCATCTTTTTTCAGATCTTCAG, reverse CGTGTTAACTGCCCTAGG; (3) H93D, *T*_m_ = 61 °C, forward TTAGTAGCATCCGATTGTGCAAG, reverse ACATAAGATCCCGATCAAATAC. The SDM protocol was performed according to manufacturer's instructions. 5 μL of the KLD reaction product was used to transform 50 μL of *E*. *coli* DH5α chemically competent cells. On average, 30 single colonies were obtained per plate. Single colonies were used to inoculate 5 mL of LB media supplemented with kanamycin antibiotic and were grown overnight at 37 °C (250 rpm). Cell pellets were harvested by centrifugation (4122*g*, 10 min, 4 °C), the supernatant was discarded and plasmid DNA was extracted and purified using GeneJET Plasmid Miniprep Kit (Thermo Scientific) as per manufacturer's instructions. DNA concentration was quantified using nanodrop DeNovix DS-11+ spectrophotometer and MbQ gene mutations were confirmed by sequencing (DNA Sequencing and Services, MRC PPU).

### Production and purification of proteins

BL21 *E. coli* competent cells were individually transformed with pET29b(+) plasmid containing desired MbQ variant gene. Briefly, 50 µL of cells were thawed on ice and then gently mixed with 1 µL (150–200 ng µL^−1^) of plasmid solution. After 30 min incubation on ice, the cells were heat-shocked (42 °C, 40 s), re-cooled on ice (2 min), recovered with room temperature SOC media and incubated for 1 h at 37 °C (250 rpm). The cells were then diluted 2-fold, plated out on LB agar plates supplemented with kanamycin antibiotic and incubated overnight at 37 °C. Freshly picked single colonies were used to inoculate 100 mL of LB media supplemented with kanamycin antibiotic and left to incubate overnight at 37 °C (180 rpm). 10 mL of the culture was used to inoculate 1 L of 2xYT media supplemented with kanamycin antibiotic. The cultures were incubated at 37 °C (230 rpm) until OD600 reached 0.5. Then IPTG was added (final concentration 1 mM) and the induced cultures were incubated overnight at 25 °C (200 rpm). The cells were harvested by centrifugation (4122*g*, 20 min, 4 °C) and the pelleted cells were suspended in PBS buffer (pH 7.5). After centrifugation (4122*g*, 10 min, 4 °C) the supernatant was decanted and the cell pellets were stored at −22 °C until further use. If cell pellets were used immediately after, lysis/wash buffer (50 mM Tris/HCl, 300 mM NaCl, 10 mM imidazole, pH 8) was added. If frozen cell pellets were used, these were first fully defrosted and then lysis buffer was added. Suspended cells were lysed using sonication (2 min, 90% power, pulse 5 s on and 5 s off). Cell lysates were centrifuged (40 374*g*, 4 °C. 1 h) and the supernatant was loaded onto HisTrap HP column (5 mL; Cytiva) equilibrated with the lysis/wash buffer. His-tagged protein was eluted using elution buffer (50 mM Tris/HCl, 300 mM NaCl, 300 mM imidazole, pH 7.4). Collected protein fractions were combined and dialysed against 50 mM Tris/HCl (pH 7) overnight at 4 °C. Heme removal procedure or full reconstitution with either Fe-PPIX-Cl or Mn-PPIX-Cl was performed next as detailed below. Otherwise, protein solutions were concentrated and flash frozen using liquid N2 and stored at −80 °C until further use.

### Heme removal

Apo Mb was prepared according to a modified Teale's heme extraction method.^19^ Briefly, a solution containing roughly 50 mg of protein in 20 mL of 50 mM Tris/HCl (pH 7) was prepared on ice. The pH of the solution was adjusted to pH 2 using 1 M HCl_aq_ solution. The protein solution was then transferred to a cooled separatory funnel and an equal volume (20 mL) of butan-2-one was added. The two solutions were gently mixed by shaking and allowed to stand on ice for approximately 15 min until the aqueous and organic layers have separated. The bottom aqueous layer containing apo Mb was collected, dialysed against 5 L of deionised water at 4 °C for 5 hours and then against 5 L of 50 mM Tris/HCl (pH 7) overnight at 4 °C.

### General procedure for preparation of Fe-PPIX-Cl and Mn-PPIX-Cl reconstituted proteins

To a 30–50 μM solution of apo Mb in 50 mM Tris/HCl (pH 7) on ice was added 1.2 molar equivalent of Fe-PPIX-Cl or Mn-PPIX-Cl complex (2 mM, DMSO) in a dropwise manner while gently stirring. The solution was left to incubate for 20 minutes, concentrated to approximately 1 mL using Amicon Ultra-15 centrifugal filters (10 kDa molecular weight cut-off) and passed through PD-10 desalting column packed with Sephadex G-25 resin equilibrated with 50 mM Tris/HCl (pH 7). Fractions containing protein solution were combined and stored at 4 °C until further use. The same procedure was applied to attain full heme occupancy in Mb proteins. We note that reconstitution of MbQ H93D scaffold with either Fe-PPIX-Cl or Mn-PPIX-Cl cofactor was partial. For catalysis, reconstituted proteins were transferred to KPi buffer (pH 7) using PD-10 columns.

### NMR spectroscopy

^1^H and ^13^C NMR spectra were recorded on Bruker Avance III 500 MHz spectrometer at 298 K. All chemical shifts are reported in parts per million (ppm). ^1^H and ^13^C NMR spectra were referenced relative to SiMe_4_ through the resonance of employed deuterated solvent proteo-impurity of the solvent: CD_3_OD (3.31 ppm), CDCl3 (7.26 ppm) for ^1^H NMR spectra (500 MHz); CD_3_OD (49.00 ppm), CDCl_3_ (77.16 ppm) for ^13^C NMR spectra (126 MHz).

### Native mass spectrometry

Native nanoESI-MS analysis of both apo and cofactor reconstituted WT Mb and MbQ variant samples was performed using Synapt G2 (Waters) equipped with TriVersa NanoMate nanoelectrospray infusion robot (Advion Biosciences). Generally, the following instrument conditions were applied: nanoelectrospray voltage 1.6 kV and gas pressure 0.6 psi; sampling cone 40 V; extraction cone 0 V; and source temperature 100 °C. All samples were measured at *ca.* 30 µM concentration in 50 mM ammonium acetate solution.

### UV-vis spectroscopy

UV-vis absorbance spectra were recorded on Varian Cary 50 spectrophotometer with a 1 cm path-length quartz cuvette. Concentration of Fe-PPIX-protein complexes was determined using *ε*_Soret_ = 188 000 M^−1^ cm^−1^ and that of Mn-PPIX-protein complexes using *ε*_376_ = 95 000 M^−1^ cm^−1^.

### CD spectroscopy

Circular dichroism spectra and analysis of thermal stability were performed using Applied Photophysics Chirascan VX spectropolarimeter equipped with Quantum Northwest TC 1 Temperature Controller with Peltier-controlled cuvette holder. Protein samples were first buffer exchanged into 10 mM potassium phosphate buffer (pH 8), filtered through 0.22 µm filter and centrifuged (12 000*g*, 10 min at 4 °C). A 200 µL quartz cuvette with 0.05 cm path-length was used to analyse protein samples at a concentration of *ca.* 0.2 mg mL^−1^. Circular dichroism scans for secondary structure content were performed at 20 °C and were measured at from 190 to 260 nm. Thermal melt experiments were performed at 222 nm and measured from either 20 to 94 °C with 2 min equilibration time allowed at each measured point every 1 °C.

### ICP measurements

Analysis of at least two independently prepared protein samples was performed using ICP-MS Single Quad Agilent 7900 and Multi-quad Agilent 8900.

### Protein film voltammetry

All protein and metal complex films were prepared and their potentials measured under N_2_ atmosphere in a glovebox (Belle, ≥2 ppm O_2_) at 25 °C using a glass cell with a water jacket. Pyrolytic graphite (PG) electrode was used as a working electrode with all samples, saturated calomel electrode (SCE) was used as a reference electrode and Pt wire was used as a counter electrode throughout. PG working electrodes were made inhouse using epoxy resin coating and had a total surface area of 0.03 cm^2^. All protein film voltammetry experiments were run in buffer mix solution (recipe given below) at either pH 4, 6, 7, 8 or 10 and contained 100 mM NaCl as a supporting electrolyte, unless stated otherwise. All obtained redox potential values were measured relative to SCE and then converted *vs.* SHE at 25 °C: *E*_SHE_ = *E*_SCE_ + 0.241 V.^40^ All electrochemistry data was collected using PalmSens4 potentiostat and PSTrace software. Calibration of the reference electrode was performed using 10 mM ferrocenemethanol solution.^40^ For the preparation of proteins films, solutions of reconstituted protein in 50 mM Tris/HCl (pH 7) were either thawed from frozen stocks or prepared freshly as detailed above. In general, protein solutions were diluted with mix buffer (pH 7) and used at 0.5 mM concentration unless stated otherwise. A 10 mM solution of DDAB in Milli-Q water was prepared using ultrasonication, as previously reported in the literature.^25^ In cases where Nafion D-521 was used, it was used as obtained from commercial suppliers (5 mM). PG working electrode was polished with 2500 grit sandpaper before preparation of fresh protein-DDAB films. 2 µL of protein solution was first added onto the working electrode. After letting it dry for ∼1 hour under N_2_ atmosphere, 2 µL of 10 mM DDAB solution was added on top and the film was left to dry overnight under the same conditions. A blank measurement before each run where protein or cofactor films were analysed was performed using no film and/or DDAB film only. All protein film voltammetry experiments were performed using buffer mix solutions (15 mM MES, HEPES, TAPS, CHES, NaOAc and 100 mM NaCl) at specified pH and scan rate. Metal complexes were analysed in 25 mM KPi buffer (pH 7) with 50 mM NaBr acting as electrolyte. All buffers were thoroughly degassed under N_2_ atmosphere before use. In the experiments where cell buffer solution pH was varied, the pH was set to either pH 4, 6, 7, 8 or 10, as detailed. For varied scan rate experiments, the scan rate was varied from 0.02 V s^−1^ to 1 V s^−1^, unless stated otherwise. Baseline-subtraction function in the CV analysis for both anodic and cathodic redox peaks was performed using Origin software.

### Fitting of electrochemistry data

Following background subtraction using spine-curve fitting and noise removal using fast Fourier transform, the reductive and oxidation redox waves of the Mn system CVs were fitted using the “fit-adsorbed” routine in QSoas. Initially, *n*-values were fixed to 1 and the best fit *E* and *Γ* values were found. Then the *n*-values were relaxed and the procedure was repeated to obtain best-fit *E*, *Γ* and *n*.

### Cyclopropanation activity study

All reactions were carried out at a 500 μL scale using vials equipped with magnetic stir bars. In a standard reaction set up, 20 μM catalyst was first reduced using 10 mM Na_2_S_2_O_4_ and then mixed with 10 mM styrene and 40 mM EDA in 100 mM KPi (pH 7). Each reaction mixture contained 6% v/v of DMF and those prepared using protein-free metal cofactor solutions contained additional 1% v/v of DMSO. Each reagent was degassed by bubbling Ar before bringing it to the glovebox and all reactions were assembled inside a glovebox under N_2_ atmosphere. Reactions were run at 25 °C for 18 hours. Reactions were quenched with 5 M HCl_aq_, followed by addition of internal standard (dodecane). Products were extracted using 4 × 200 μL of ethyl acetate and combined fractions were analysed using GC-FID on either Rxi-5 ms or chiral Rt-bDEXm columns. Protein containing catalyst concentration was determined using reported extinction coefficients (see above).

### GC-FID analysis

Previously reported separation methods were modified and applied to analyse extracted solutions from styrene cyclopropanation reactions.^38^ Non-chiral analysis using Rxi-5 ms capillary column (30 m × 0.32 mm ID, 1.0 μm df): 1.0 μL injection volume; injector *T* = 250 °C; detector *T* = 300 °C; column *T* = 90 °C (start), increased to 250 °C at 12.0 °C min^−1^, held for 2 min, increased to 300 °C at 30 °C min^−1^ and held for 1 min. Total run time = 18.0 min. Chiral analysis for *trans* cyclopropane product separation using Rt-bDEXm capillary column (30 m, 0.25 mm ID, 0.25 μm df): 1.0 μL injection volume; injector *T* = 250 °C; detector *T* = 250 °C; column *T* = 60 °C (start), increased to 165 °C at 0.5 °C min^−1^, increased to 210 °C at 25 °C min^−1^ and held for 5 min. Total run time = 216.8 min.

## Author contributions

EV performed experimental work, data analysis and interpretation, wrote the initial draft and prepared the figures. PRM and AGJ conceived the project idea, acquired the funding and contributed to the experimental work, data analysis and interpretation. EV, PRM and AGJ conceptualized the project and prepared the final version of the manuscript. All authors have read and approved the final version of the manuscript for submission.

## Conflicts of interest

The authors declare no competing financial interest.

## Supplementary Material

DT-OLF-D6DT00266H-s001

## Data Availability

The data supporting this article have been included as part of the supplementary information (SI). Supplementary information is available. See DOI: https://doi.org/10.1039/d6dt00266h.
