# Supplementary material for: Direct electrochemistry as a mechanistic tool for studying engineered myoglobins: implications on carbene transferase activity
Source: Dalton Trans. 2026 Jun 16. Online ahead of print. doi: 10.1039/d6dt00266h (PMC13288710; doi:10.1039/d6dt00266h)

Supplementary Information (SI) for Dalton Transactions.
This journal is © The Royal Society of Chemistry 2026

Supporting Information for

Direct Electrochemistry as a Mechanistic Tool for Studying Engineered Myoglobins: Implications on Carbene Transferase Activity

Evelina Venckute[1], Amanda G. Jarvis*[1] and Patricia Rodríguez-Maciá*[2]

[1] EaStCHEM School of Chemistry, Joseph Black Building, University of Edinburgh, David Brewster Road, Edinburgh EH9 3FJ, United Kingdom
[2]School of Chemistry and Leicester Institute for Structural and Chemical Biology, George Porter Building, University of Leicester, University Road, Leicester LE1 7RH, United Kingdom

Amino acid sequence of His-tagged MbQ:

```
MVLSEGEWQLVLHVWAKVEADVAGHGQDILIRLFKSHPETLEKFDRFKHLKTEAEMKASEDL
KKHGVTVLTALGAILKKKGHHEAELKPLAQSHATKHKIPIKYLEFISEAIIHVLHSRHPGDF
GADAQGAMNKALELFRKDIAAKYKELGYQGGSGHHHHHH
```

DNA sequence of pET29b(+) His-tagged MbQ:

```
ATGGTTCTGTCTGAAGGTGAATGGCAGCTGGTTCTGCATGTTTGGGCTAAAGTTGAAGCTGA
CGTCGCTGGTCATGGTCAGGACATCTTGATTCGACTGTTCAAATCTCATCCGGAAATTCTGG
AAAAATTCGATGATCTGAAACATCTGAAAACTGAAGCTGAAATGAAAGCTTCTGAAGATCTG
AAAAAACATGGTGTTACCGTGTTAACTGCCCTAGGTGCTATCCTTAAGAAAAAAGGGCATCA
TGAAGCTGAGCTCAAACCGCTTGCACAATCGCATGCTACTAAACATAAGATCCCGATCAAAT
ACCTGGAATTCTTCTCTGAAGCGATCATCCATGTTCTGCATTCTAGACATCCAGGTGACTTC
GGTGCTGACGCTCAGGGTGCTATGAACAAAGCTCTGGAGCTGTTCCGTAAAGATATCGCTGC
TAAGTACAAAGAACTGGGTTACCAGGGTGGCTCGGGACATCATCACCATCACCAT
```

Table S1. Native nanoESI-MS results and expression yields of Mb scaffolds used in this work. All details is given for apo-Mb with His-tag.

Scaffold	Theoretical Mass (Da)	Native nanoESI-MS Determined Mass (Da)	Yield (mg/L culture)[a]
Mb-WT	18355.1	18355.1	37
MbQ	18326.1	18325.9	>200
MbQ-His64Gly	18246.0	18247.0	199
MbQ-His64Asp	18304.0	18304.1	197
MbQ-His93Asp	18304.0	18306.9	65

[a]expressed as a mix of *holo* and *apo* protein.

Table S2. Native nanoESI-MS analysis results for Fe-PPIX and Mn-PPIX reconstituted MbQ variants.

Scaffold	Cofactor	Native nanoESI-MS Determined Mass (Da)
MbQ	Fe-PPIX	18942.3

	Mn-PPIX	18943.9
MbQ His64Gly	Fe-PPIX	18865.8
	Mn-PPIX	18864.5
MbQ His64Asp	Fe-PPIX	18918.4
	Mn-PPIX	18917.8
MbQ His93Asp	Fe-PPIX	18922.2
	Mn-PPIX	18920.8

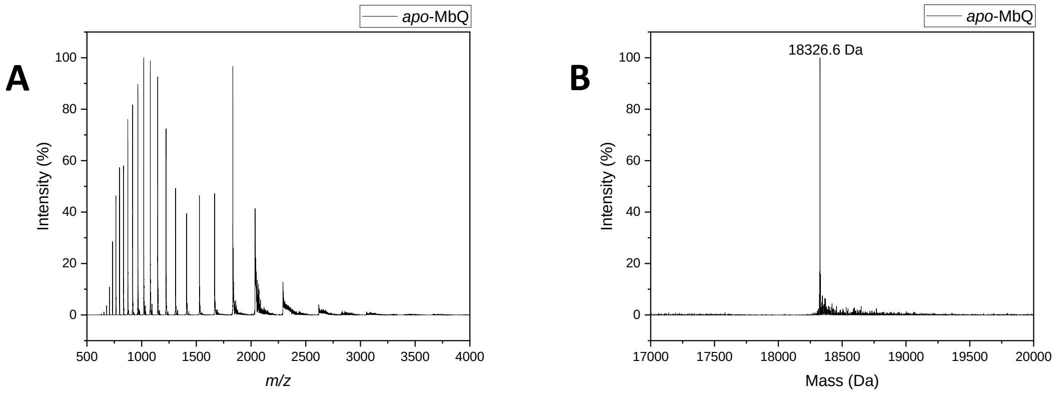

Figure S1. Native nanoESI-MS spectra of *apo*-MbQ. A) Spectra showing charge states. B) Deconvoluted spectra showing obtained experimental mass.

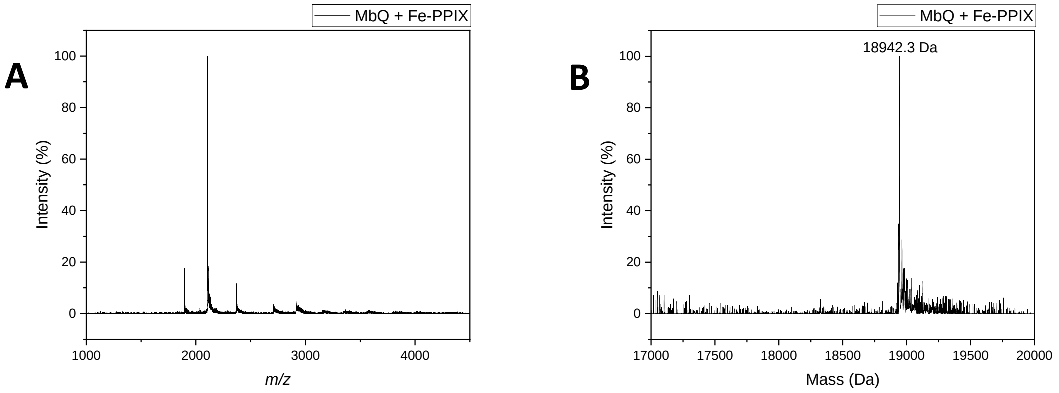

Figure S2. Native nanoESI-MS spectra of MbQ reconstituted with Fe-PPIX. A) Spectra showing charge states. B) Deconvoluted spectra showing obtained experimental mass for Fe-PPIX bound MbQ complex.

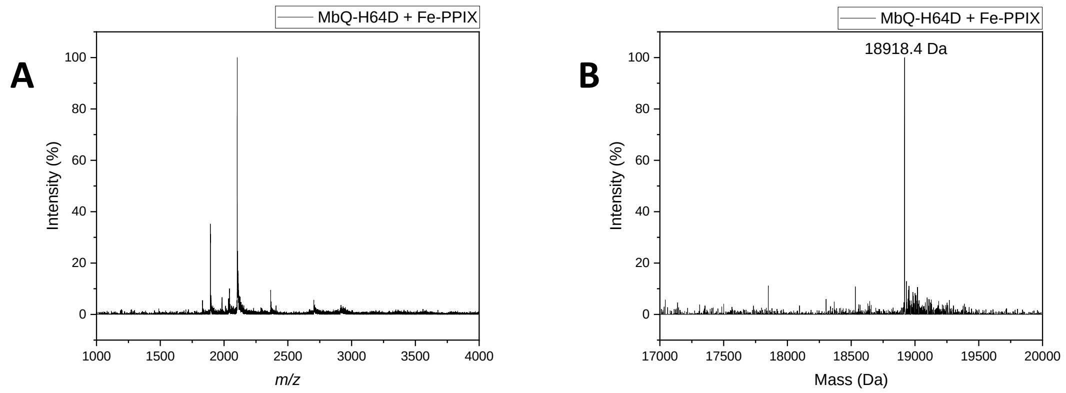

Figure S3. Native nanoESI-MS spectra of MbQ-H64D reconstituted with Fe-PPIX. A) Spectra showing charge states. B) Deconvoluted spectra showing obtained experimental mass for Fe-PPIX bound MbQ-H64D complex.

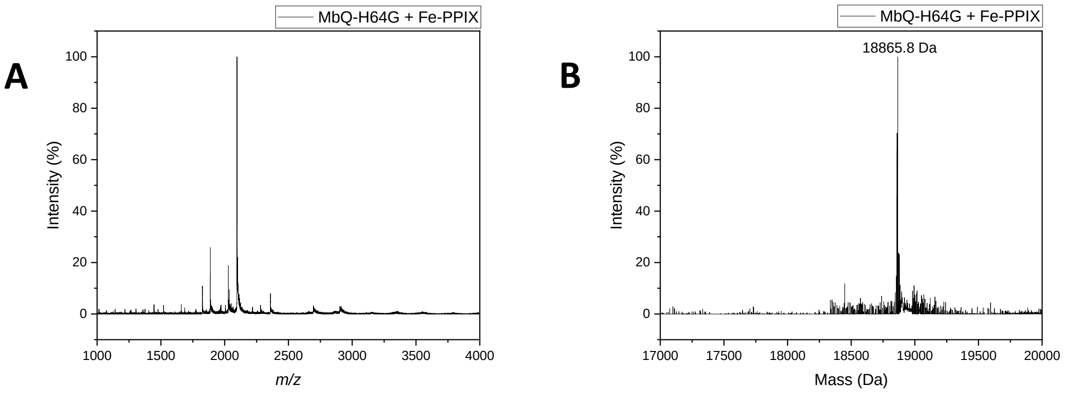

Figure S4. Native nanoESI-MS spectra of MbQ-H64G reconstituted with Fe-PPIX. A) Spectra showing charge states. B) Deconvoluted spectra showing obtained experimental mass for Fe-PPIX bound MbQ-H64G complex.

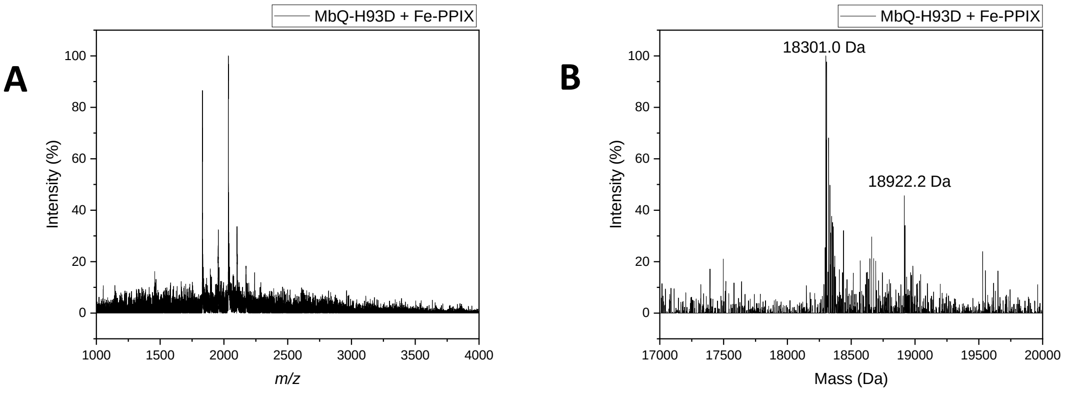

Figure S5. Native nanoESI-MS spectra of MbQ-H93D reconstituted with Fe-PPIX. A) Spectra showing charge states. B) Deconvoluted spectra showing obtained experimental mass for Fe-PPIX bound MbQ-H93D complex.

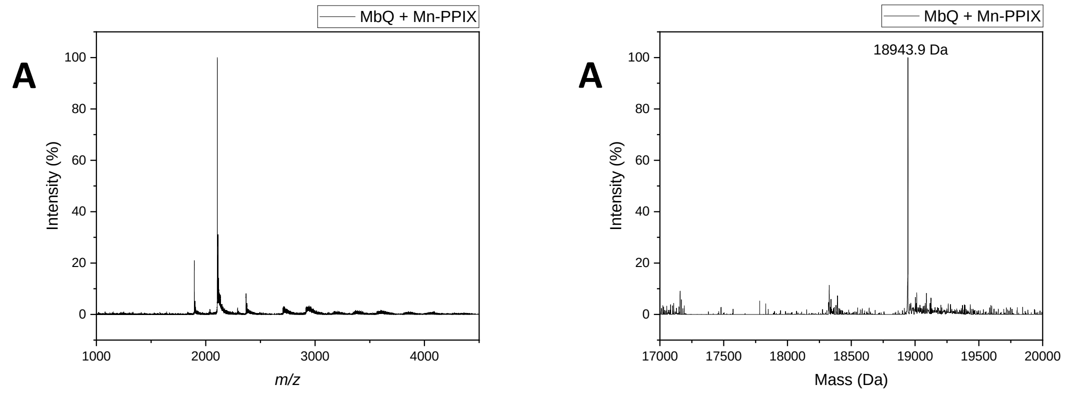

Figure S6. Native nanoESI-MS spectra of MbQ reconstituted with Mn-PPIX. A) Spectra showing charge states. B) Deconvoluted spectra showing obtained experimental mass for Mn-PPIX bound MbQ complex.

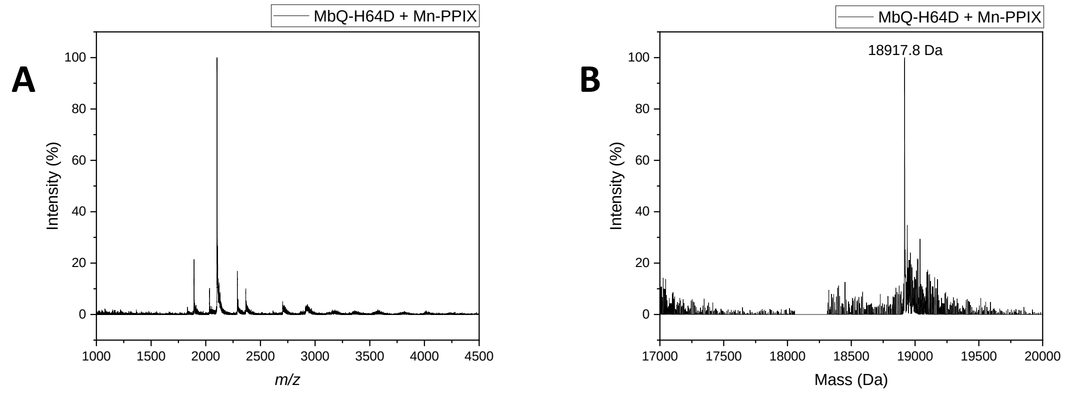

Figure S7. Native nanoESI-MS spectra of MbQ-H64D reconstituted with Mn-PPIX. A) Spectra showing charge states. B) Deconvoluted spectra showing obtained experimental mass for Mn-PPIX bound MbQ-H64D complex.

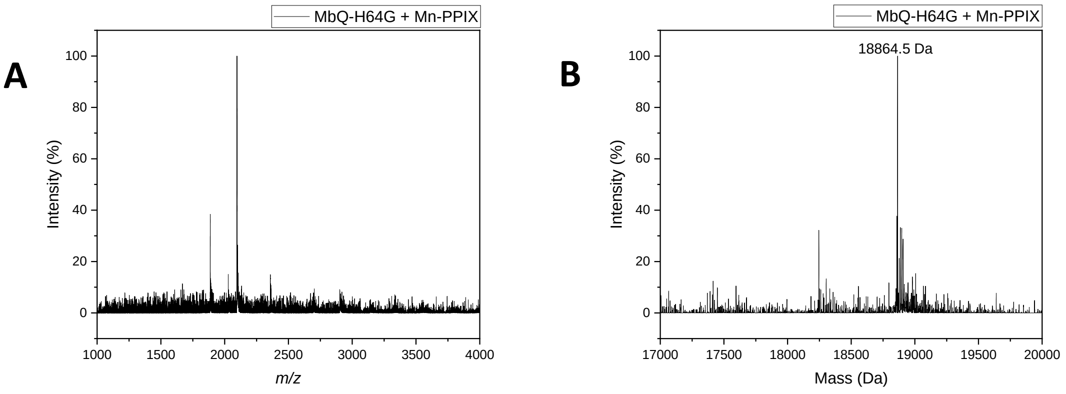

Figure S8. Native nanoESI-MS spectra of MbQ-H64G reconstituted with Mn-PPIX. A) Spectra showing charge states. B) Deconvoluted spectra showing obtained experimental mass for Mn-PPIX bound MbQ-H64G complex.

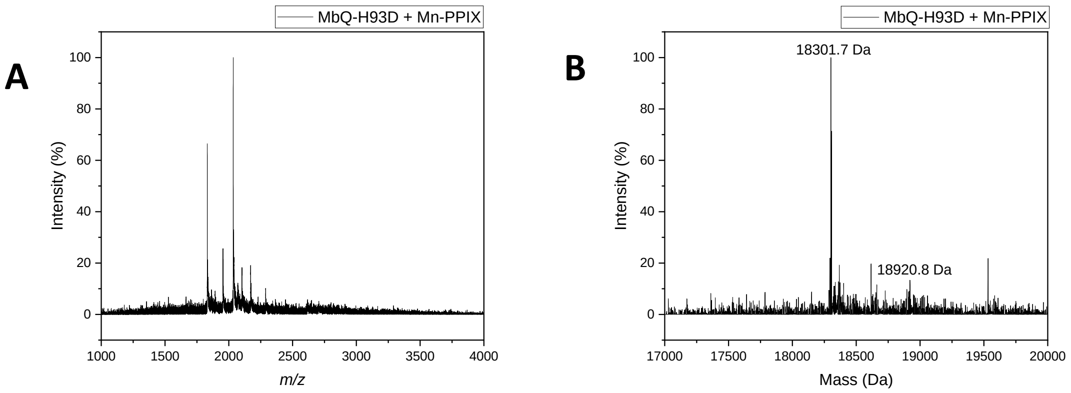

Figure S9. Native nanoESI-MS spectra of MbQ-H93D reconstituted with Mn-PPIX. A) Spectra showing charge states. B) Deconvoluted spectra showing obtained experimental mass for Mn-PPIX bound MbQ-H93D complex.

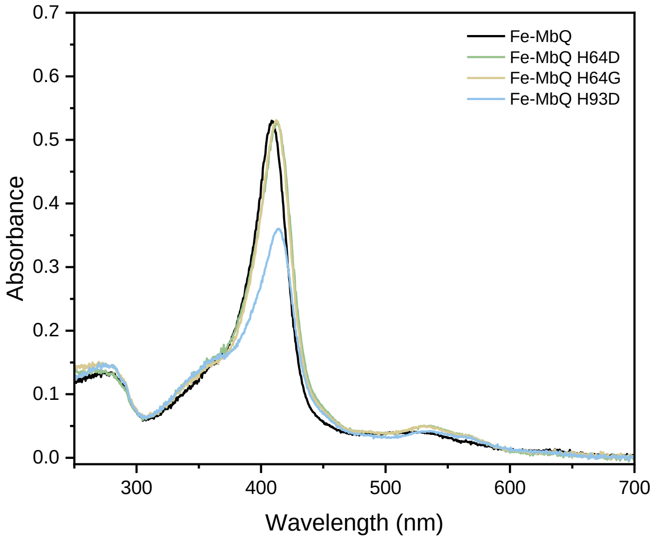

Figure S10. UV-vis absorbance spectra of heme-bound MbQ variants (ca. 3 μM, c lower for Fe-MbQ H93D) in 50 mM Tris/HCl (pH 7).

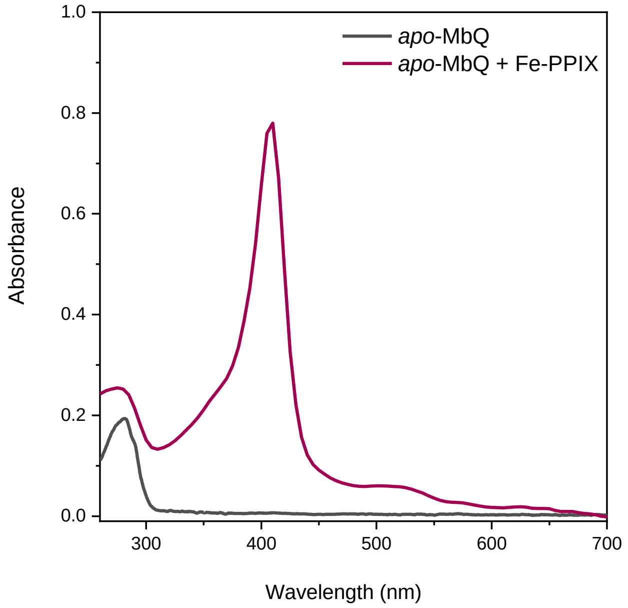

Figure S11. UV-vis absorbance trace of heme-bound MbQ (red line) and *apo* MbQ (black line) in 50 mM Tris/HCl (pH 7). *Apo* MbQ was obtained after heme extraction using Teale's method.

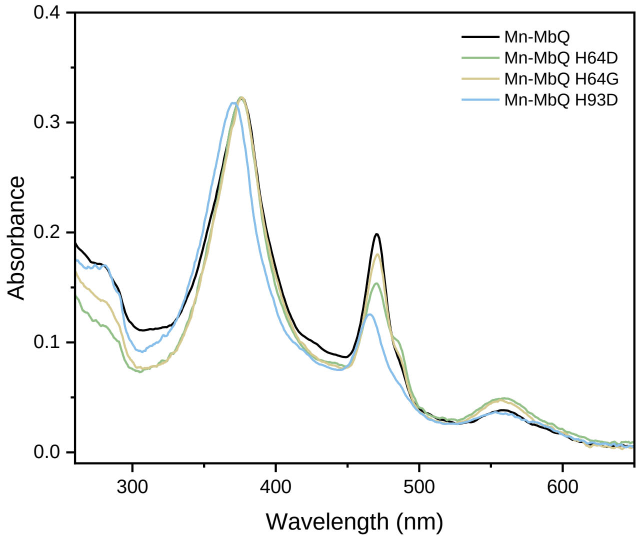

Figure S12. UV-vis absorbance spectra of Mn-PPIX-bound MbQ variants (ca. 3 μM) in 50 mM Tris/HCl (pH 7).

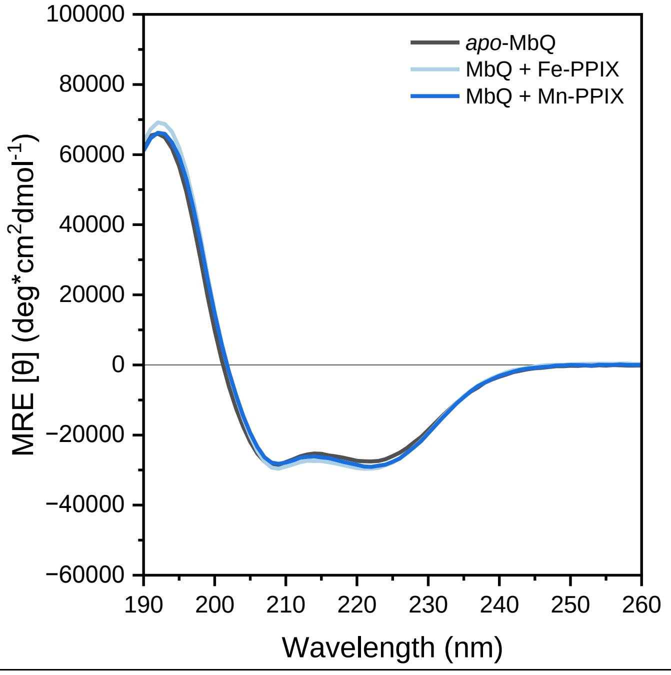

Protein system	T_m (°C)
apo-MbQ	60.8
MbQ Fe-PPIX	72.0
MbQ Mn-PPIX	78.2

Figure S13. Circular dichroism spectra and thermal analysis results for *apo*-MbQ (black line), MbQ reconstituted with Fe-PPIX (light blue line) and with Mn-PPIX (dark blue line).

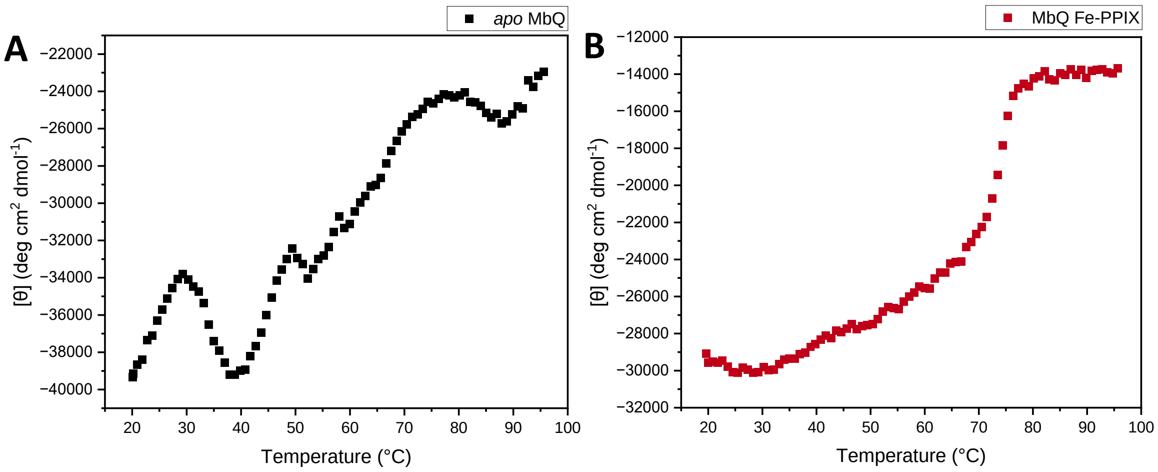

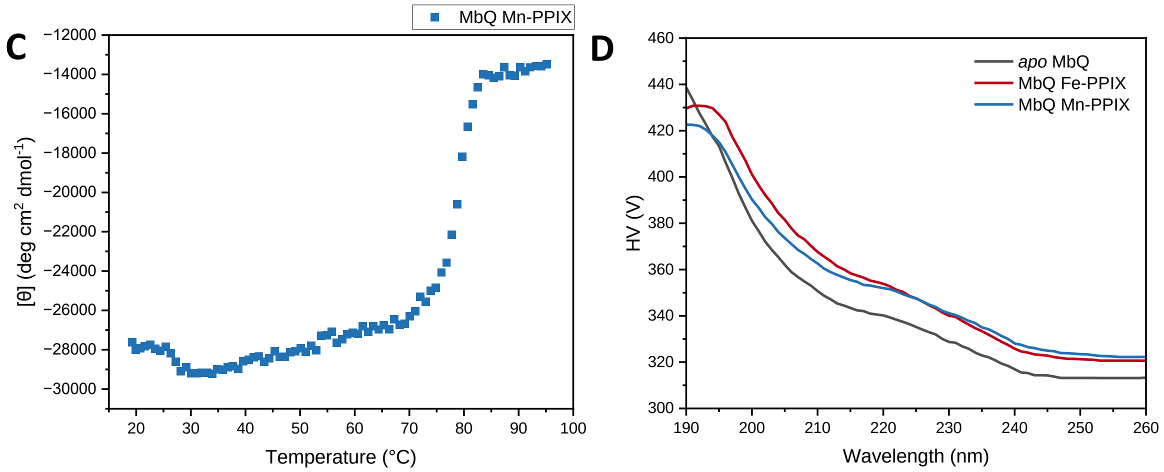

Figure S14. Thermal melt plots of A) *apo* MbQ, B) MbQ reconstituted with Fe-PPIX and C) MbQ reconstituted with Mn-PPIX determined at 222 nm. D) High voltage control plots.

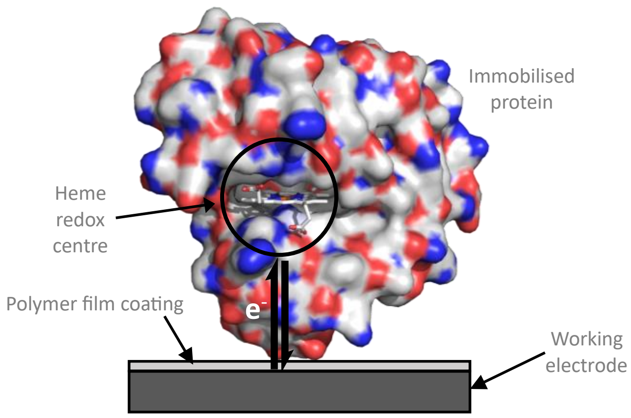

Figure S15. Direct electrochemistry measurement of WT Mb system adsorbed on a pyrolytic graphite electrode surface using polymer film coating. The film coating on the electrode surface positions the redox centre in a close proximity to the electrode surface for efficient electron transfer. WT Mb has a mixed surface charge (positive charge – blue, negative charge – red).

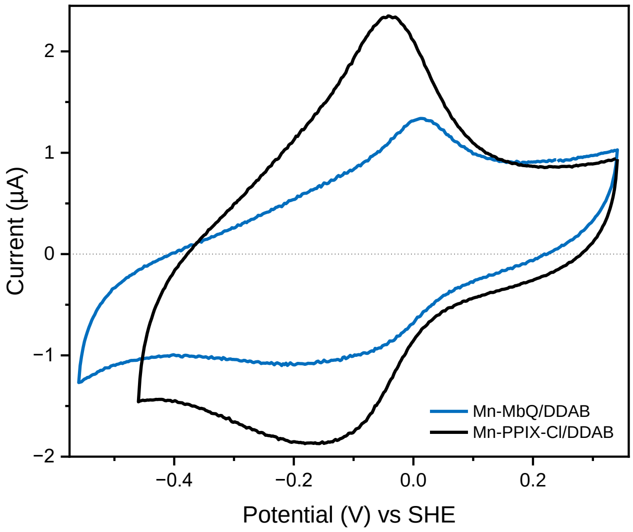

Figure S16. Overlapped CVs of Mn-MbQ and protein-free Mn-PPIX-Cl cofactor measured immobilised with DDAB surfactant on PG electrode surface. Measurements were performed under N_2 atmosphere at 25 °C in buffer mix (pH 7) solution using Mn-MbQ sample and in 25 mM KPi and 50 mM NaBr (pH 7) using Mn-PPIX-Cl sample at 0.1 V/s scan rate.

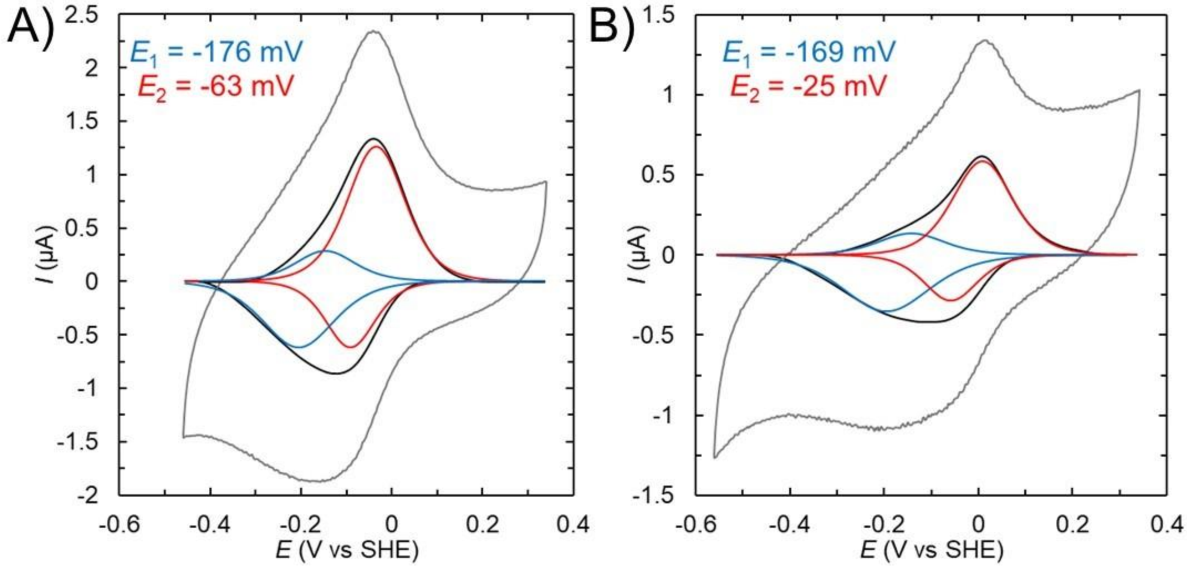

Figure S17. Fitting of the of the protein-free Mn-PPIX-Cl (A) and Mn-MbQ (B) CVs using QSoas CVs measured immobilised with DDAB surfactant on a PGE. Measurements were performed under N_2 atmosphere at 25 °C in buffer mix (pH 7) solution for Mn-MbQ and in 25 mM KPi and 50 mM NaBr (pH 7) for Mn-PPIX-Cl, both at 0.1 V/s scan rate. The low potential component in both cases is very similar (E = –169 mV vs E = –176 mV) while the high potential component is quite different (E = –63 mV in the free cofactor sample, while E = –25 mV in the Mn-MbQ sample). Best-fit parameters are listed in the table below. For both Mn-PPIX-Cl and Mn-MbQ (B), the n-values are around 0.5, and the peak-to-peak separations are around 60 mV. For species 1 in both cases, the oxidative peak has a smaller area than the reductive peak, while

for species 2, the oxidative peak has a larger area than the reductive peak. The total area of both oxidative peaks and of both reductive peaks are quite similar.

Table S3. Parameters obtained from the CV fitting.

Sample	Oxidative			Reductive			E_{av} (mV)	ΔE (mV)
Mn-PPIX-Cl	E (mV)	n	Γ (pmol cm^{-2})	E (mV)	n	Γ (pmol cm^{-2})		
Species 1	–147	0.59	173	–206	0.49	449	–176	58
Species 2	-34	0.60	747	–92	0.71	309	–63	57
Mn-MbQ	E (mV)	n	Γ (pmol cm^{-2})	E (mV)	n	Γ (pmol cm^{-2})	E_{av} (mV)	ΔE (mV)
Species 1	–142	0.55	88	–196	0.41	299	–169	54
Species 2	+9	0.60	345	–59	0.68	150	–25	68

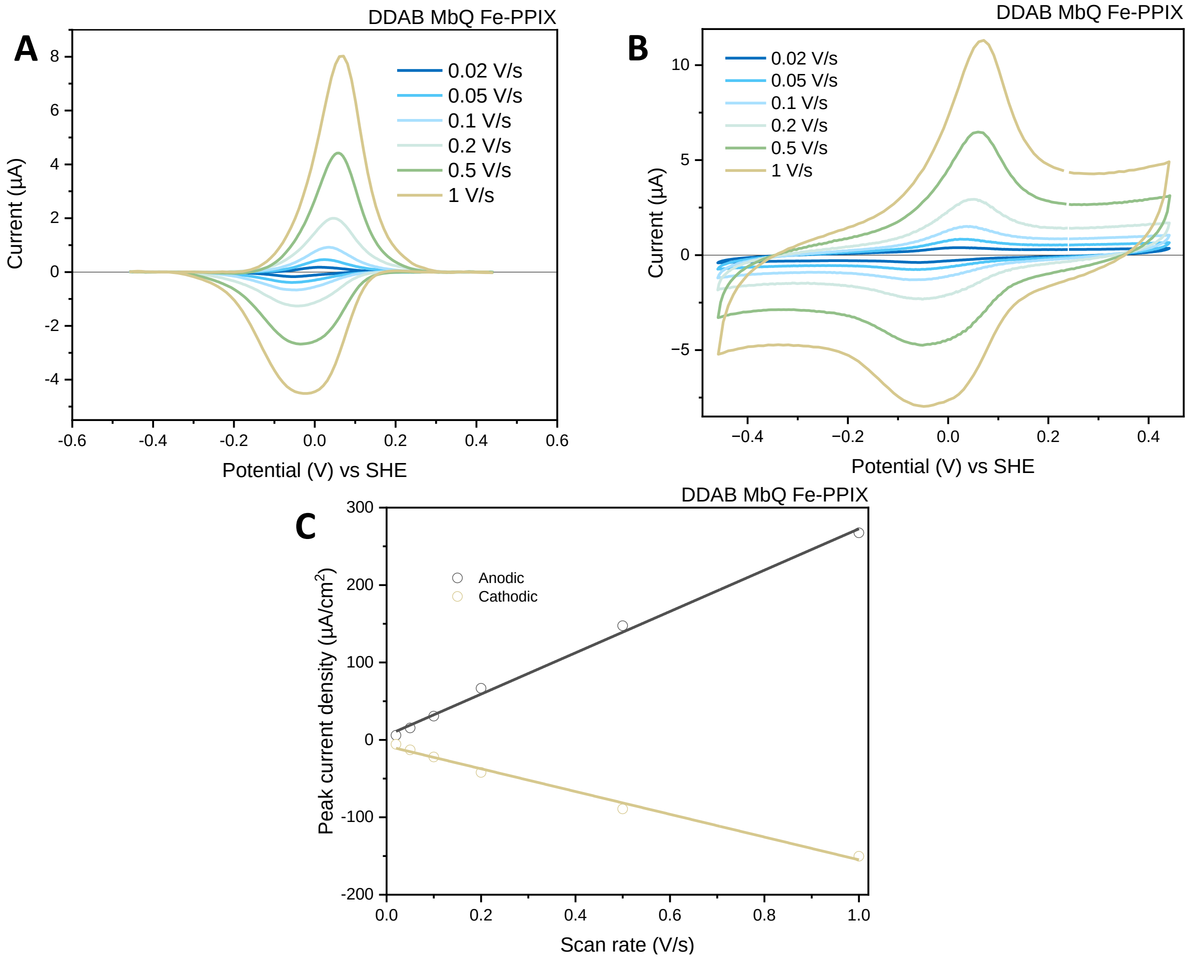

Figure S18 CVs of immobilised MbQ Fe-PPIX in DDAB films on PG electrode surface. A) Baseline subtracted and B) raw varied scan rate CVs are given along with the C) corresponding anodic and cathodic peak current density vs scan rate plot. Measurements were performed under N_2 atmosphere at 25 °C in buffer mix (pH 7) solution using 0.02-1 V/s scan rate.

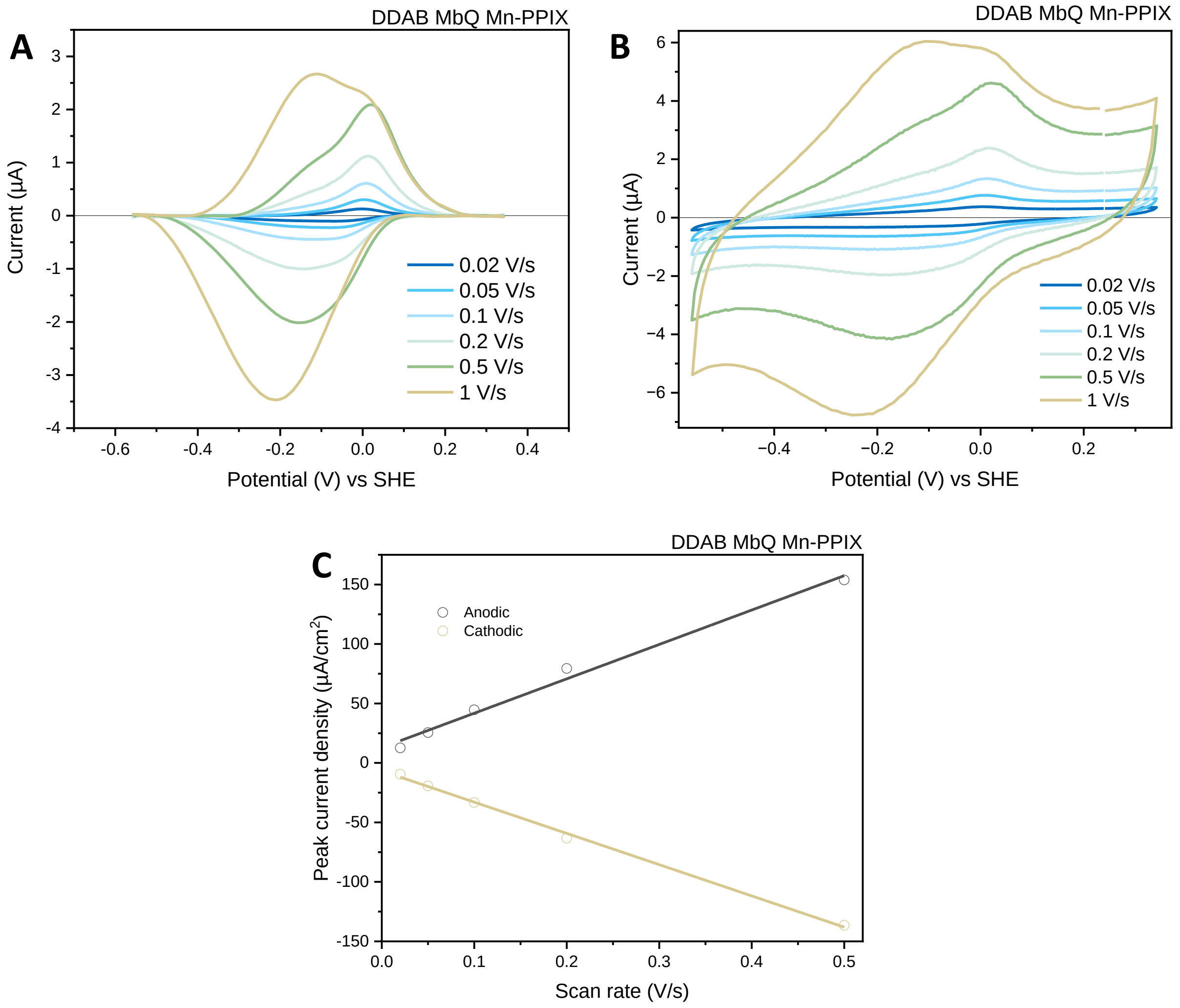

Figure S19. CVs of immobilised MbQ Mn-PPIX in DDAB films on PG electrode surface. A) Baseline subtracted and B) raw varied scan rate CVs are given along with the C) corresponding anodic and cathodic peak current density vs scan rate plot. Measurements were performed under N_2 atmosphere at 25 °C in buffer mix (pH 7) solution using 0.02-1 V/s scan rate.

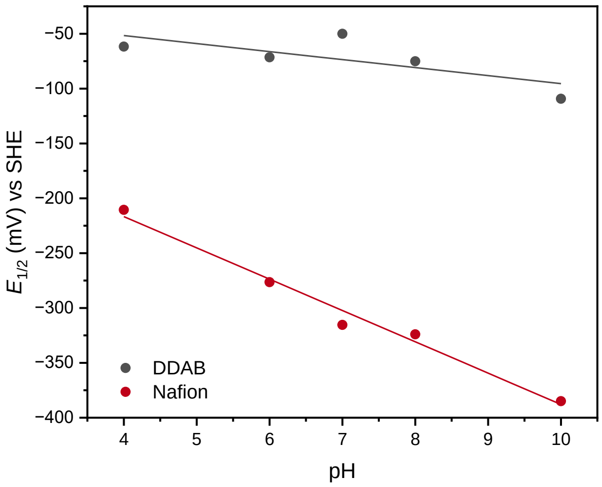

Figure S20. Varied pH effect on $E_{1/2}$ MbQ Mn-PPIX adsorbed in either DDAB (top) or Nafion-D521 (bottom) films on PG electrode surface. Measurements were performed in buffer mix at either pH 4, 6, 7, 8 or 10 at a scan rate of 0.1 V/s at 25 °C.

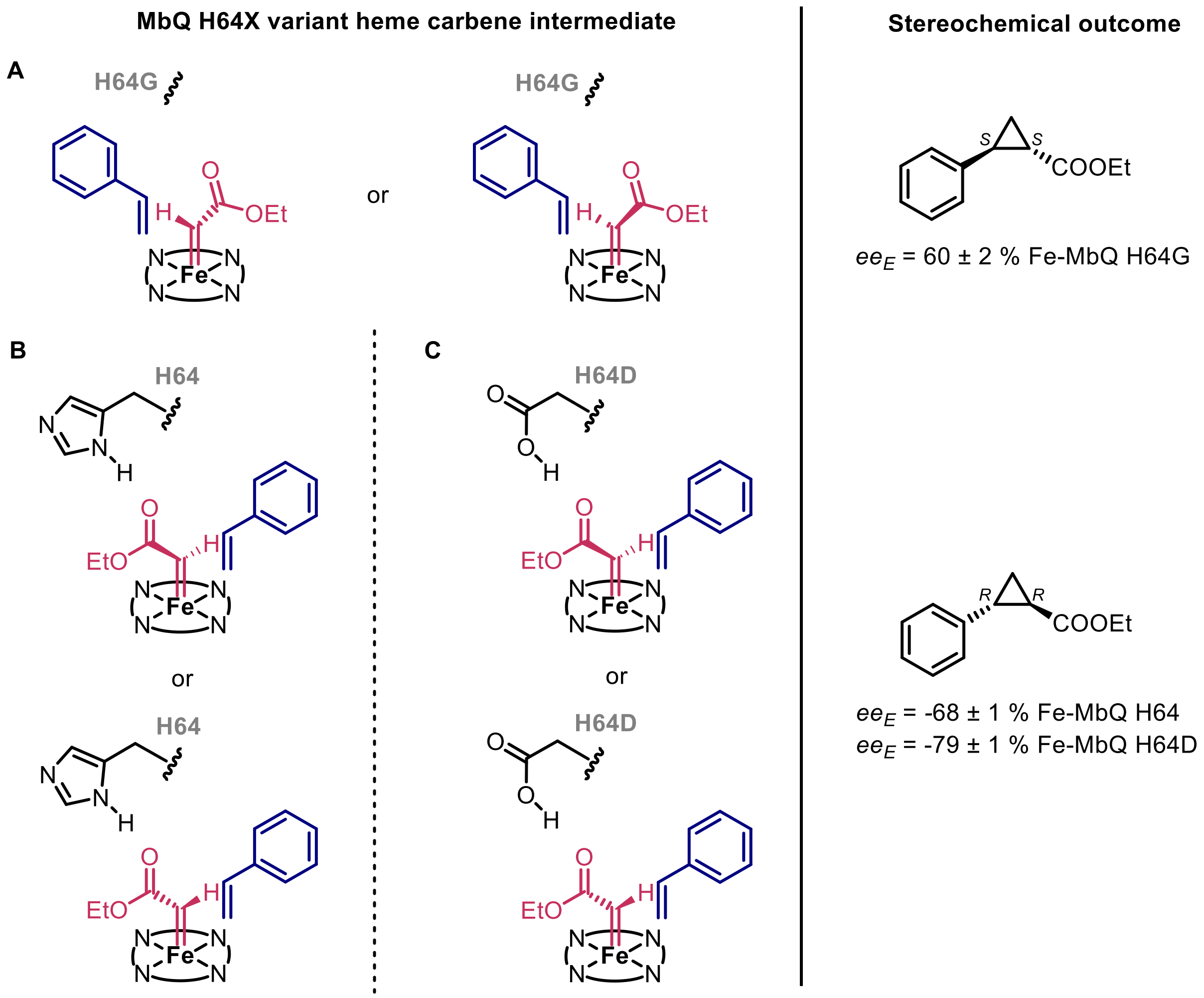

Figure S21. Schematic representation of the distal site architecture in MbQ variant scaffolds A) MbQ H64G, B) MbQ H64 and C) MbQ H64D and respective heme-carbene intermediates responsible for the reversed enantioselectivity in styrene cyclopropanation reaction.[36]

Table S4. Comparison of Mn catalyst activity in styrene cyclopropanation reaction.[a]

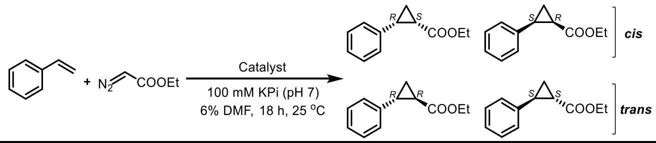

Entry	Catalyst	TTN[b]	de_E (%)[c]	ee_E (%)[d]
1	Mn-PPIX-Cl	-[e]	-	-
2	Mn-MbQ	0.24 ± 0.02	>99	–13
3	Mn-MbQ H64D	0.98 ± 0.07	>99	–72
4	Mn-MbQ H64G	0.93 ± 0.03	>99	66
5	Mn-MbQ H93D	0.41 ± 0.02	>99	4

[a]General conditions: [catalyst] = 20 μM, [styrene] = 10 mM, [EDA] = 40 mM, [$Na_2S_2O_4$] = 10 mM in 100 mM KPi buffer (pH 7) with 6% v/v DMF under N_2 atmosphere at 25 °C for 18 h. Reactions setup using Fe-PPIX-Cl and Mn-PPIX-Cl contained 1 % v/v DMSO. [b]Total turnover number, n≥3 replicates, TTN=([cis products]+[trans products])/([catalyst]). [c]Diastereomeric excess, n≥3, calculated for the trans products where de=([trans]-[cis])/([trans]+[cis])×100. [d]Enantiomeric excess, n≥3 replicates, calculated for (1S,2S)-cyclopropane in the trans products where ee_E=([1*S*,2*S*]-[1*R*,2*R*])/([1*S*,2*S*]+[1*R*,2*R*])×100. [e]No activity.

Further ICP-MS analysis of selected apo Mb samples (see below) obtained after heme-extraction and dialysis procedures revealed trace amounts of Fe. Thus, we cannot rule out that residual Fe was catalytically active and responsible for detected minimal activity (TTN <1) detected in catalytic reactions where Mn-MbQ variant samples were present.

Table S5. ICP-MS measurements for residual Fe content determination in apo proteins after heme extraction and dialysis.[a]

**Protein Scaffold**	**Fe Content in Protein Sample (%)**	
	**Run 1**	**Run 2**
apo MbQ	0.14	0.37
apo MbQ H64G	0.24	0.39

[a]data from two independent measurements. Percentage based on molar concentration.

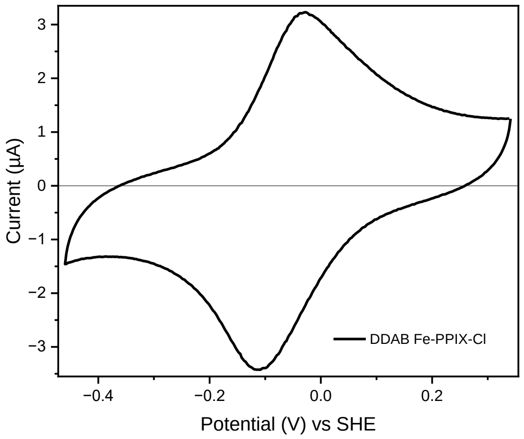

Figure S22. CV of Fe-PPIX-Cl cofactor in DDAB film. Measurements were performed in 25 mM KPi and 50 mM NaBr (pH 7) at a scan rate of 0.1 V/s at 25 °C.

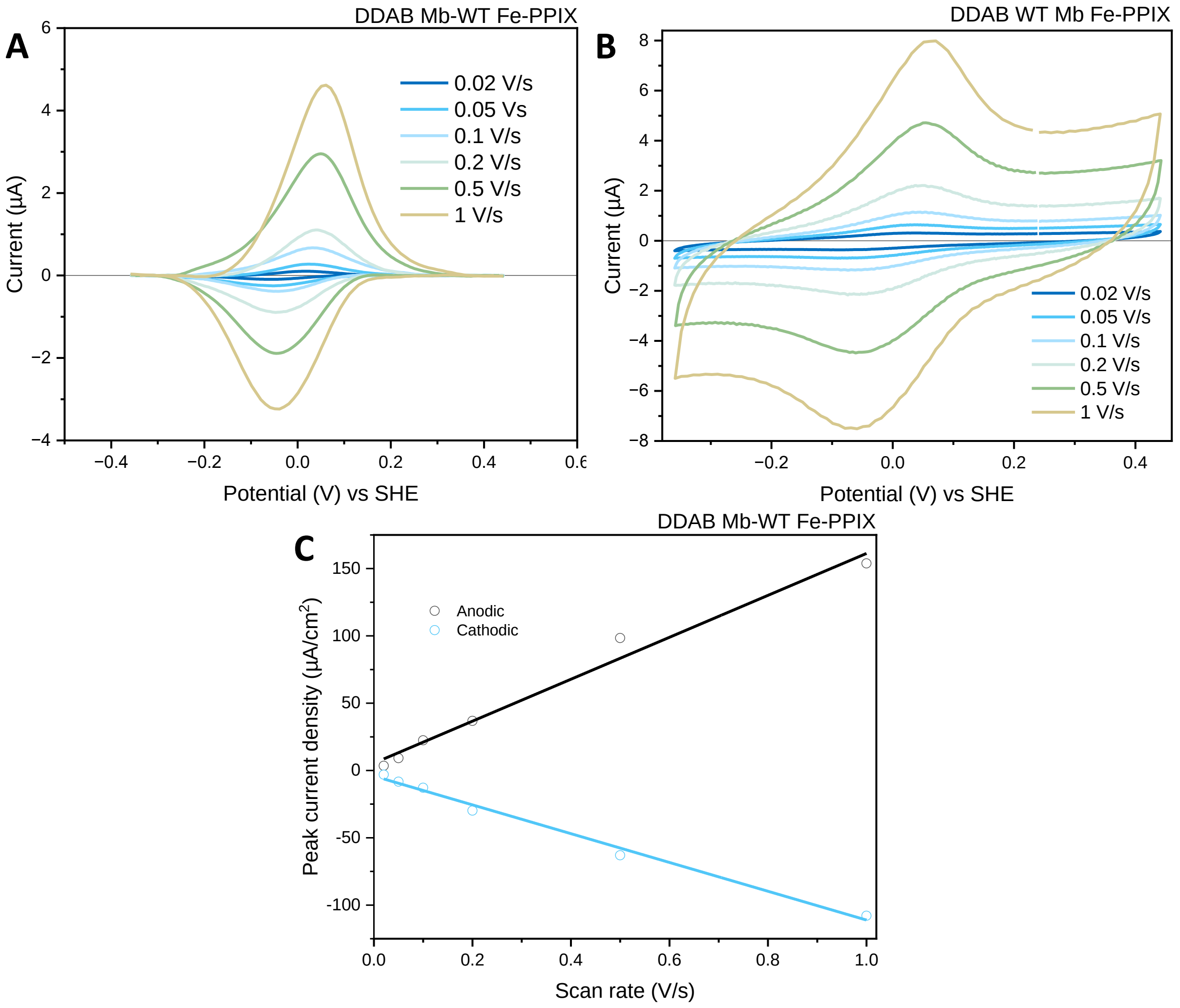

Figure S23. CVs of immobilised horse heart Mb Fe-PPIX in DDAB films on PG electrode surface. A) Baseline subtracted and B) raw varied scan rate CVs are given along with the C) corresponding anodic and cathodic peak current density vs scan rate plot. Measurements were performed under N_2 atmosphere at 25 °C in buffer mix (pH 7) solution using 0.02-1 V/s scan rate.

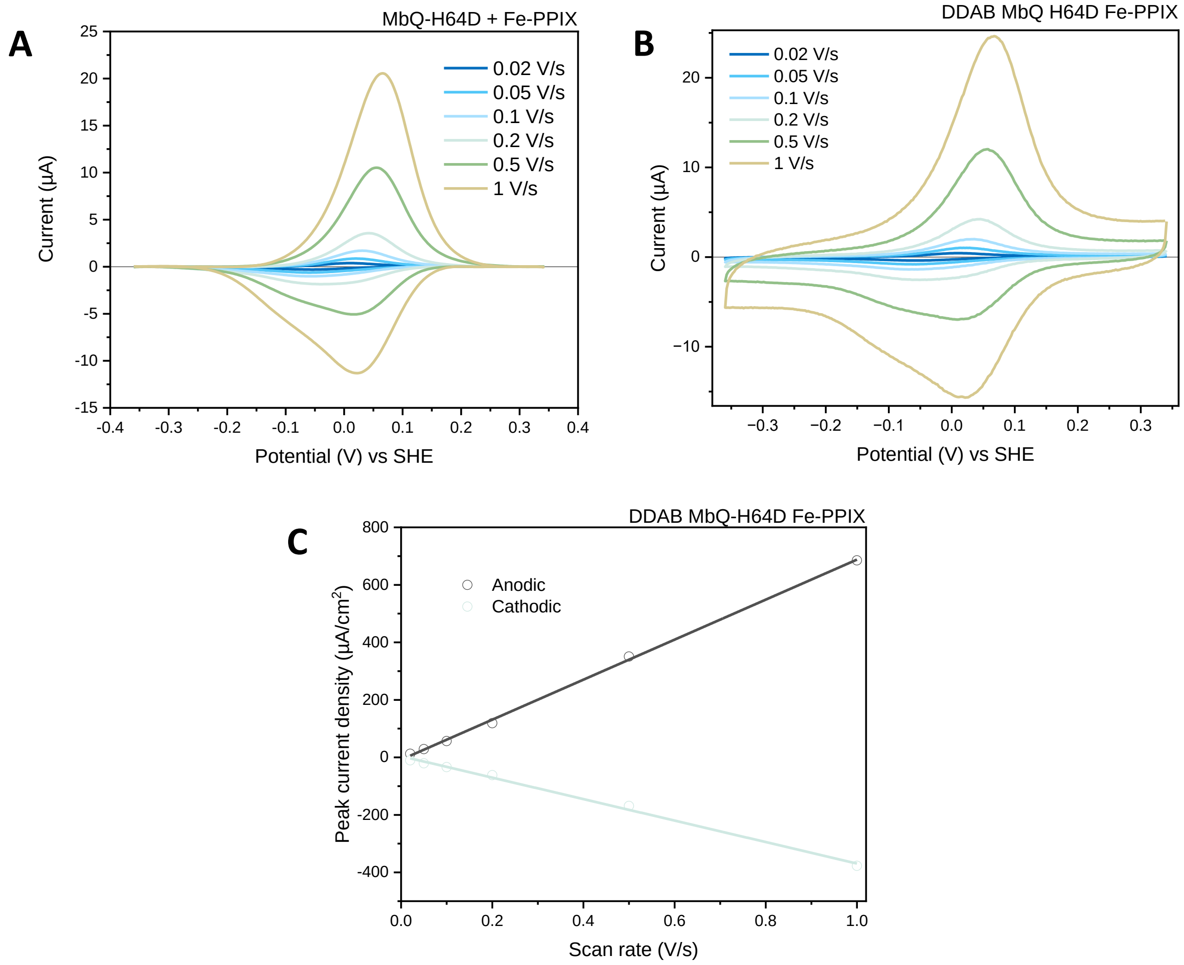

Figure S24. CVs of immobilised MbQ H64D Fe-PPIX in DDAB films on PG electrode surface. A) Baseline subtracted and B) raw varied scan rate CVs are given along with the C) corresponding anodic and cathodic peak current density vs scan rate plot. Measurements were performed under N_2 atmosphere at 25 °C in buffer mix (pH 7) solution using 0.02-1 V/s scan rate.

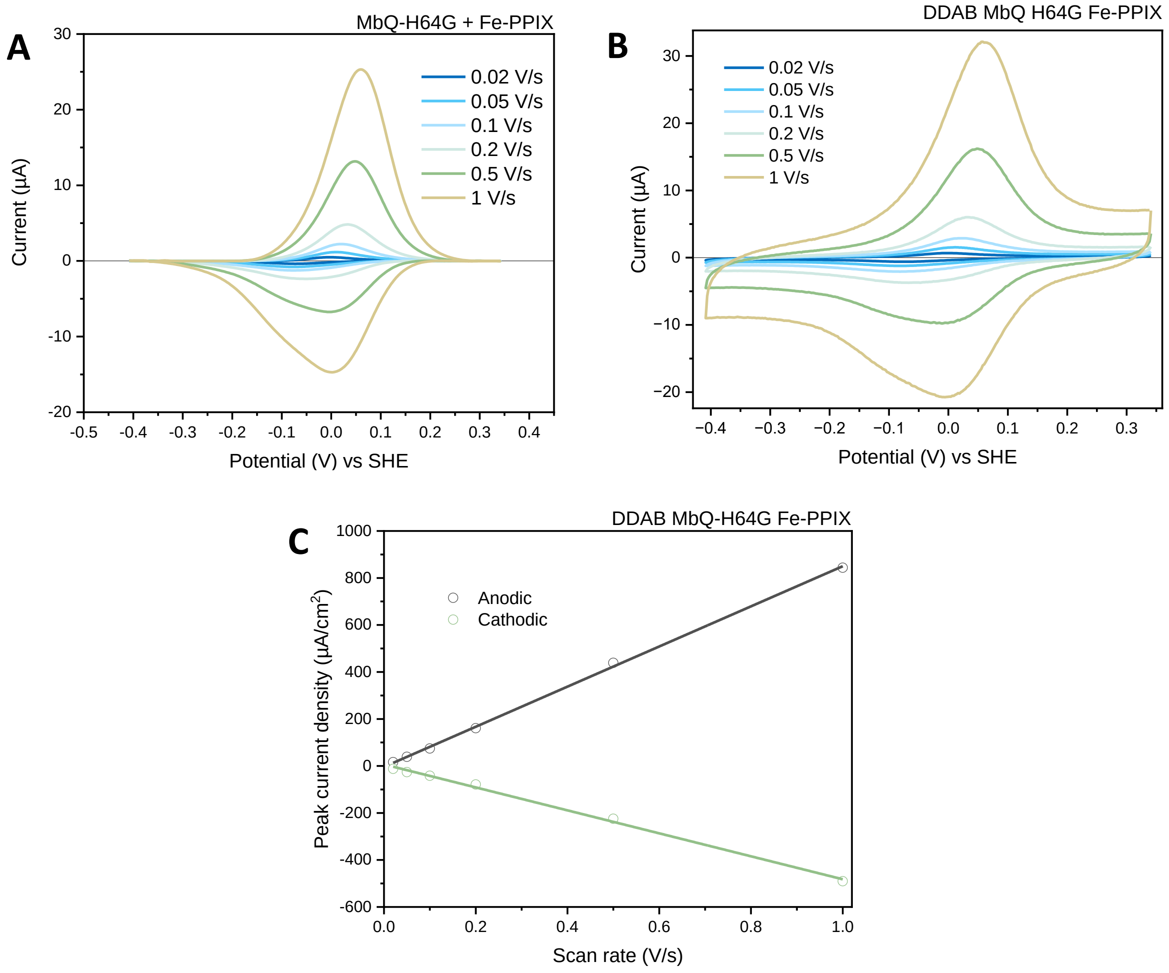

Figure S25. CVs of immobilised MbQ H64G Fe-PPIX in DDAB films on PG electrode surface. A) Baseline subtracted and B) raw varied scan rate CVs are given along with the C) corresponding anodic and cathodic peak current density vs scan rate plot. Measurements were performed under N_2 atmosphere at 25 °C in buffer mix (pH 7) solution using 0.02-1 V/s scan rate.

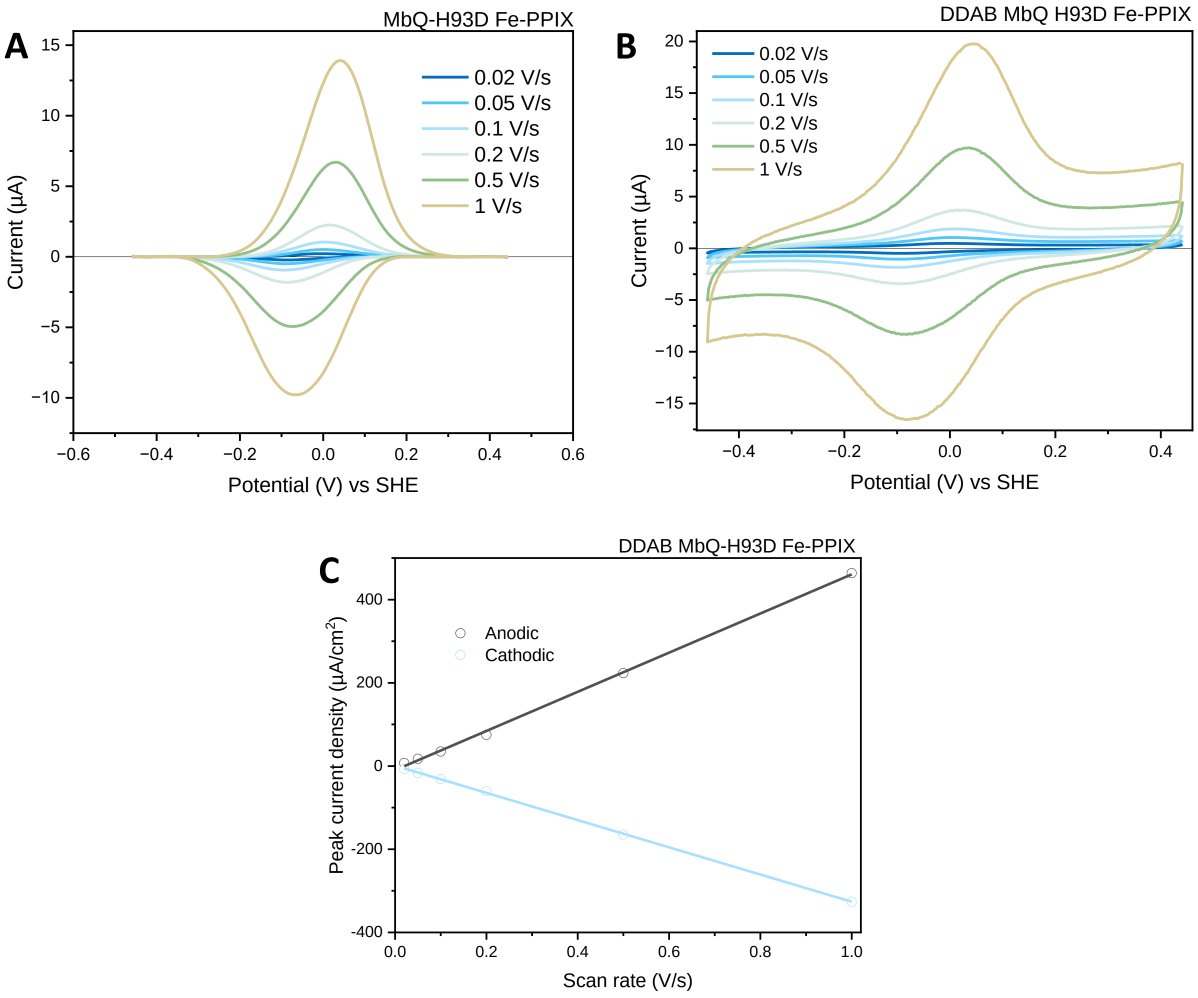

Figure S26. CVs of immobilised MbQ H93D Fe-PPIX in DDAB films on PG electrode surface. A) Baseline subtracted and B) raw varied scan rate CVs are given along with the C) corresponding anodic and cathodic peak current density vs scan rate plot. Measurements were performed under N_2 atmosphere at 25 °C in buffer mix (pH 7) solution using 0.02-1 V/s scan rate.

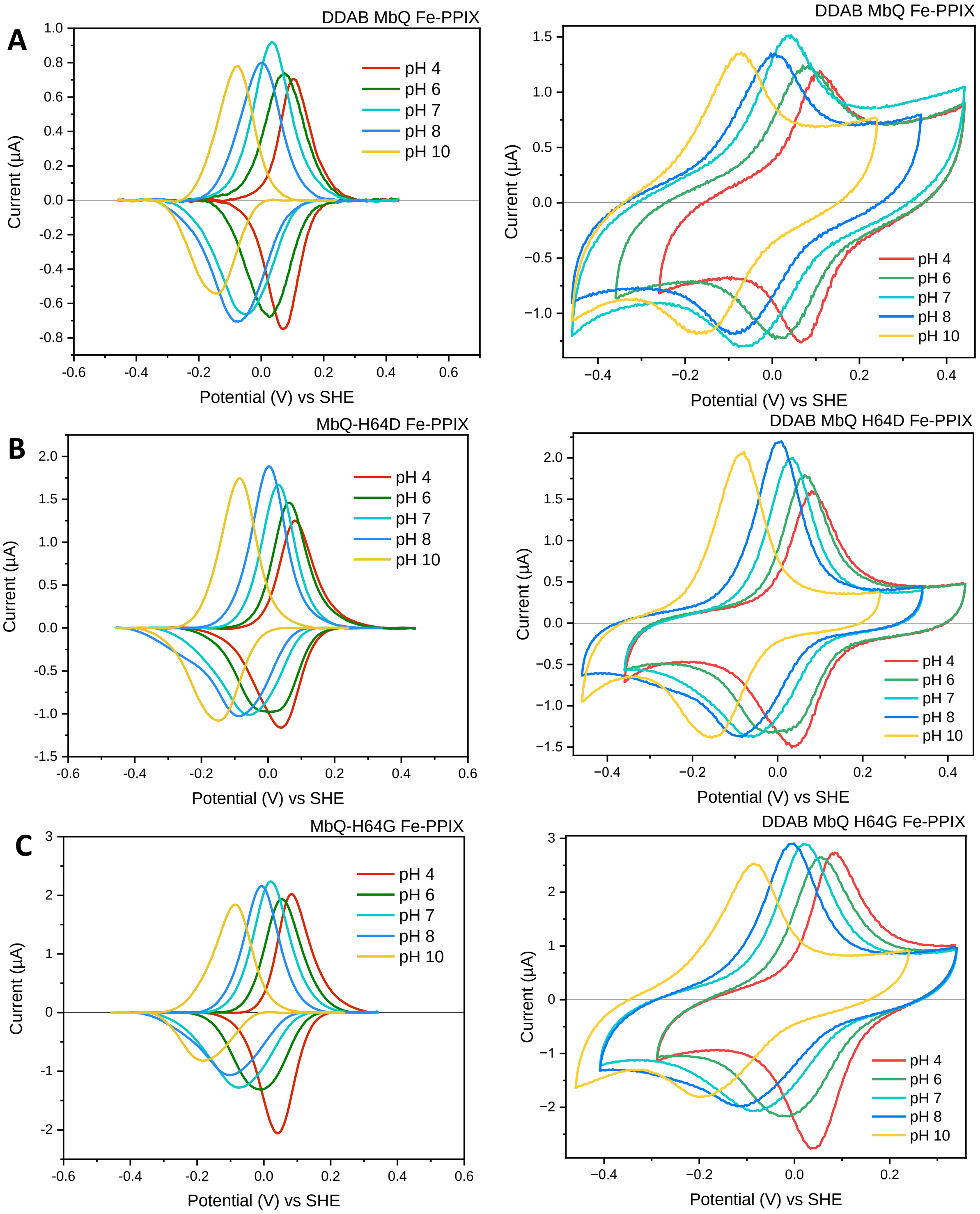
A
DDAB MbQ Fe-PPIX
Current (μA)
Potential (V) vs SHE
pH 4
pH 6
pH 7
pH 8
pH 10
DDAB MbQ Fe-PPIX
Current (μA)
Potential (V) vs SHE
pH 4
pH 6
pH 7
pH 8
pH 10
B
MbQ-H64D Fe-PPIX
Current (μA)
Potential (V) vs SHE
pH 4
pH 6
pH 7
pH 8
pH 10
DDAB MbQ H64D Fe-PPIX
Current (μA)
Potential (V) vs SHE
pH 4
pH 6
pH 7
pH 8
pH 10
C
MbQ-H64G Fe-PPIX
Current (μA)
Potential (V) vs SHE
pH 4
pH 6
pH 7
pH 8
pH 10
DDAB MbQ H64G Fe-PPIX
Current (μA)
Potential (V) vs SHE
pH 4
pH 6
pH 7
pH 8
pH 10

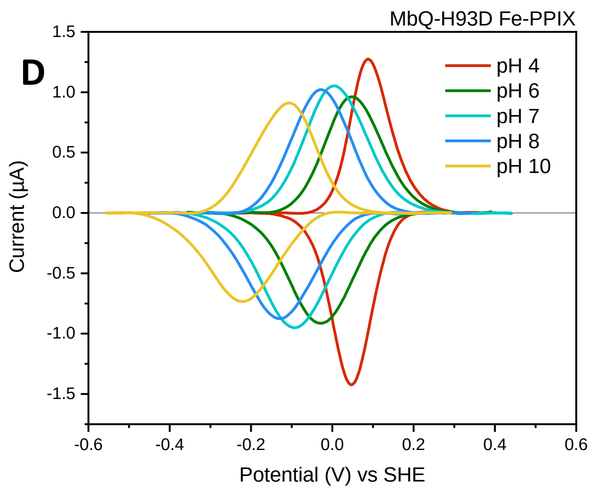

Figure S27. CVs, baseline subtracted (left) and raw (right), of A) MbQ, B) MbQ H64D, C) MbQ H64G and D) MbQ H93D bound to Fe-PPIX measured adsorbed in DDAB film on PG electrode surface. Measurements were performed under N_2 atmosphere at 25 °C in buffer mix solution at either pH 4, 6, 7, 8 or 10 at a scan rate of 0.1 V/s.

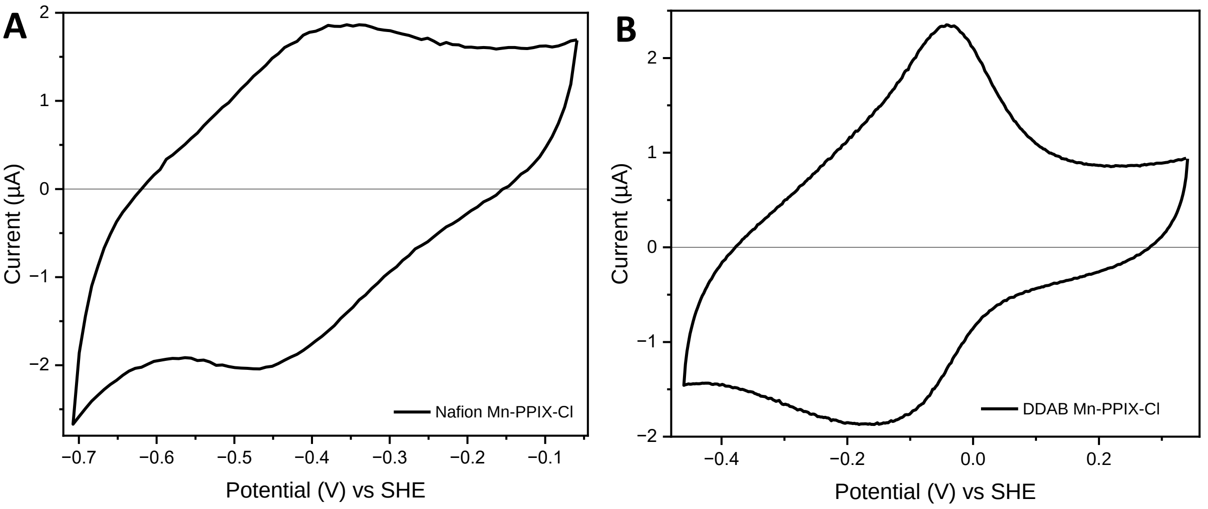

Figure S28. Cyclic voltammograms of Mn-PPIX-Cl cofactor adsorbed in either A) Nafion-D521 or B) DDAB films on PG electrode surface. Measurements were performed in 25 mM KPi and 50 mM NaBr (pH 7) at a scan rate of 0.1 V/s at 25 °C.

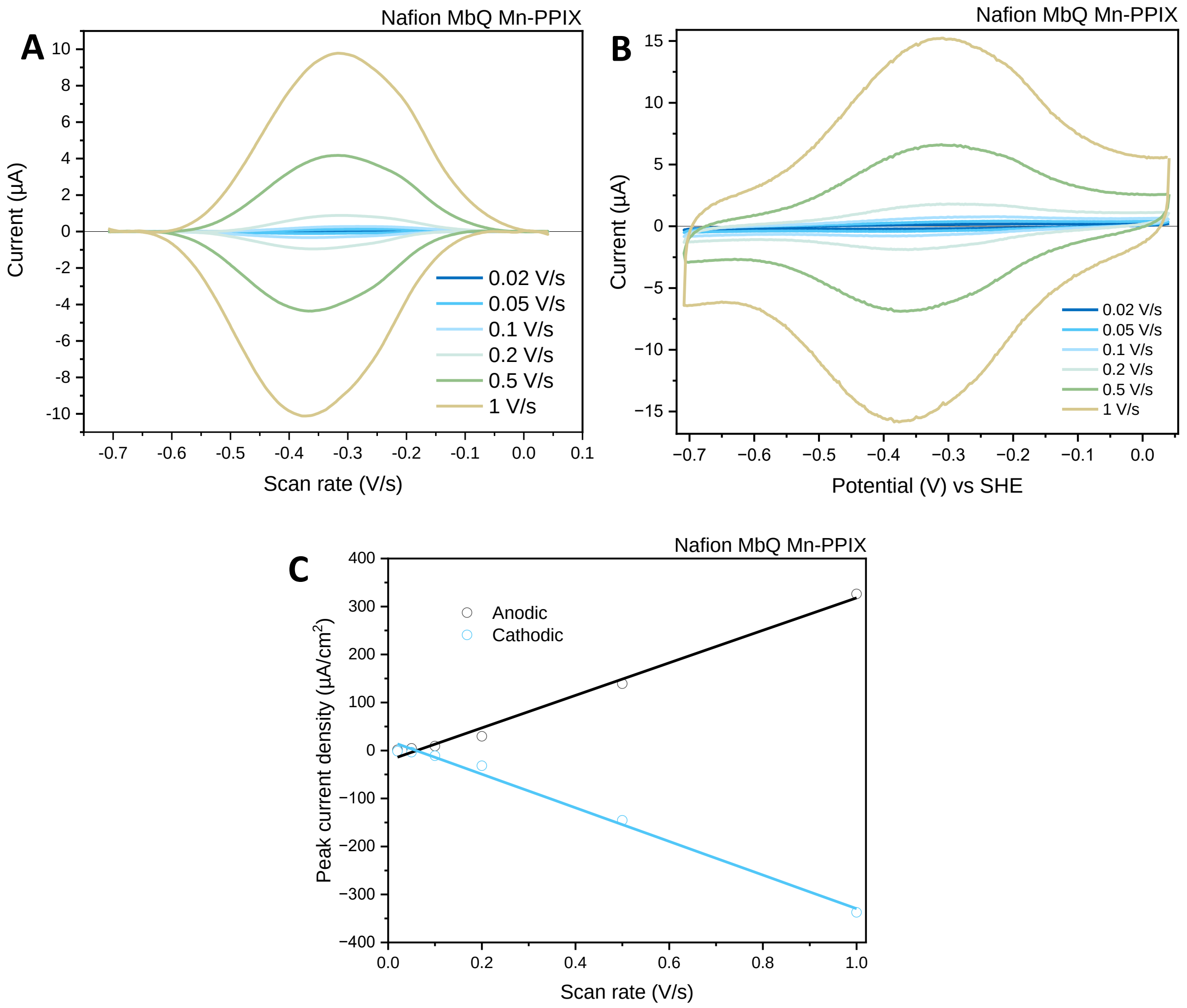

Figure S29. CVs of immobilised MbQ Mn-PPIX in Nafion-D521 films on PG electrode surface. A) Baseline subtracted and B) raw varied scan rate CVs are given along with the C) corresponding anodic and cathodic peak current density vs scan rate plot. Measurements were performed under N_2 atmosphere at 25 °C in buffer mix (pH 7) solution using 0.02-1 V/s scan rate.

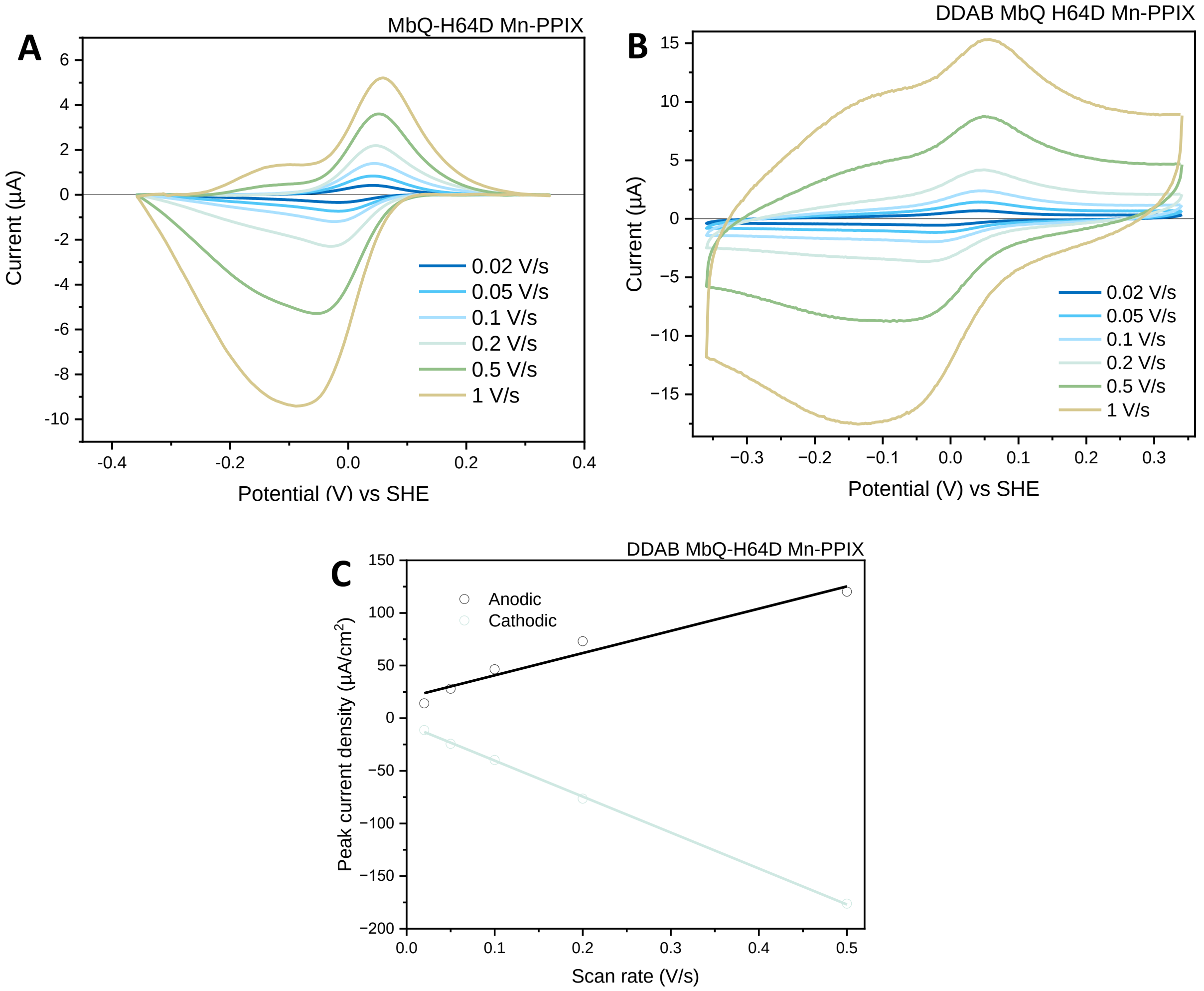

Figure S30. CVs of immobilised MbQ H64D Mn-PPIX in DDAB films on PG electrode surface. A) Baseline subtracted and B) raw varied scan rate CVs are given along with the C) corresponding anodic and cathodic peak current density vs scan rate plot. Measurements were performed under N_2 atmosphere at 25 °C in buffer mix (pH 7) solution using 0.02-1 V/s scan rate.

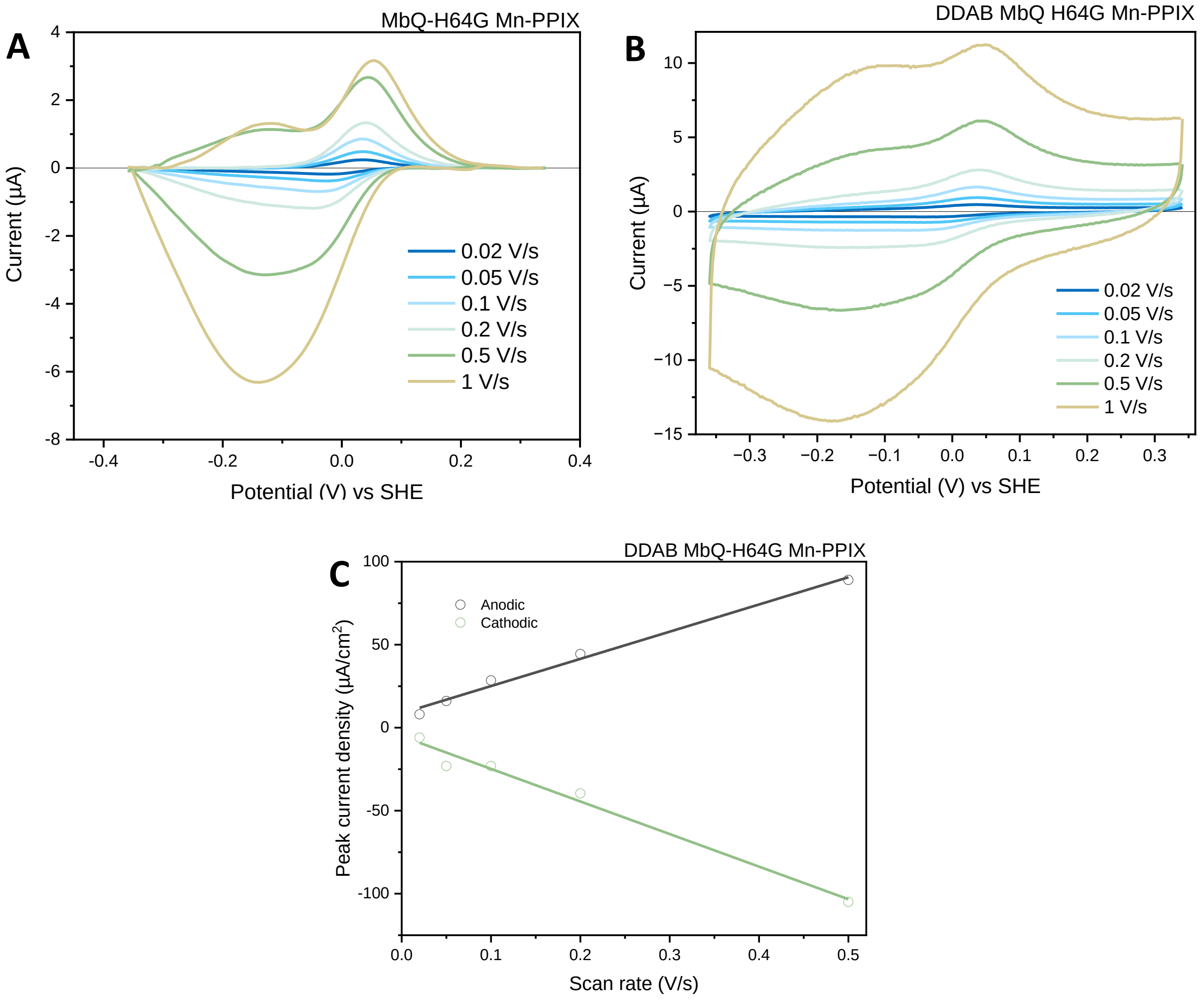

Figure S31. CVs of immobilised MbQ H64G Mn-PPIX in DDAB films on PG electrode surface. A) Baseline subtracted and B) raw varied scan rate CVs are given along with the C) corresponding anodic and cathodic peak current density vs scan rate plot. Measurements were performed under N_2 atmosphere at 25 °C in buffer mix (pH 7) solution using 0.02-1 V/s scan rate.

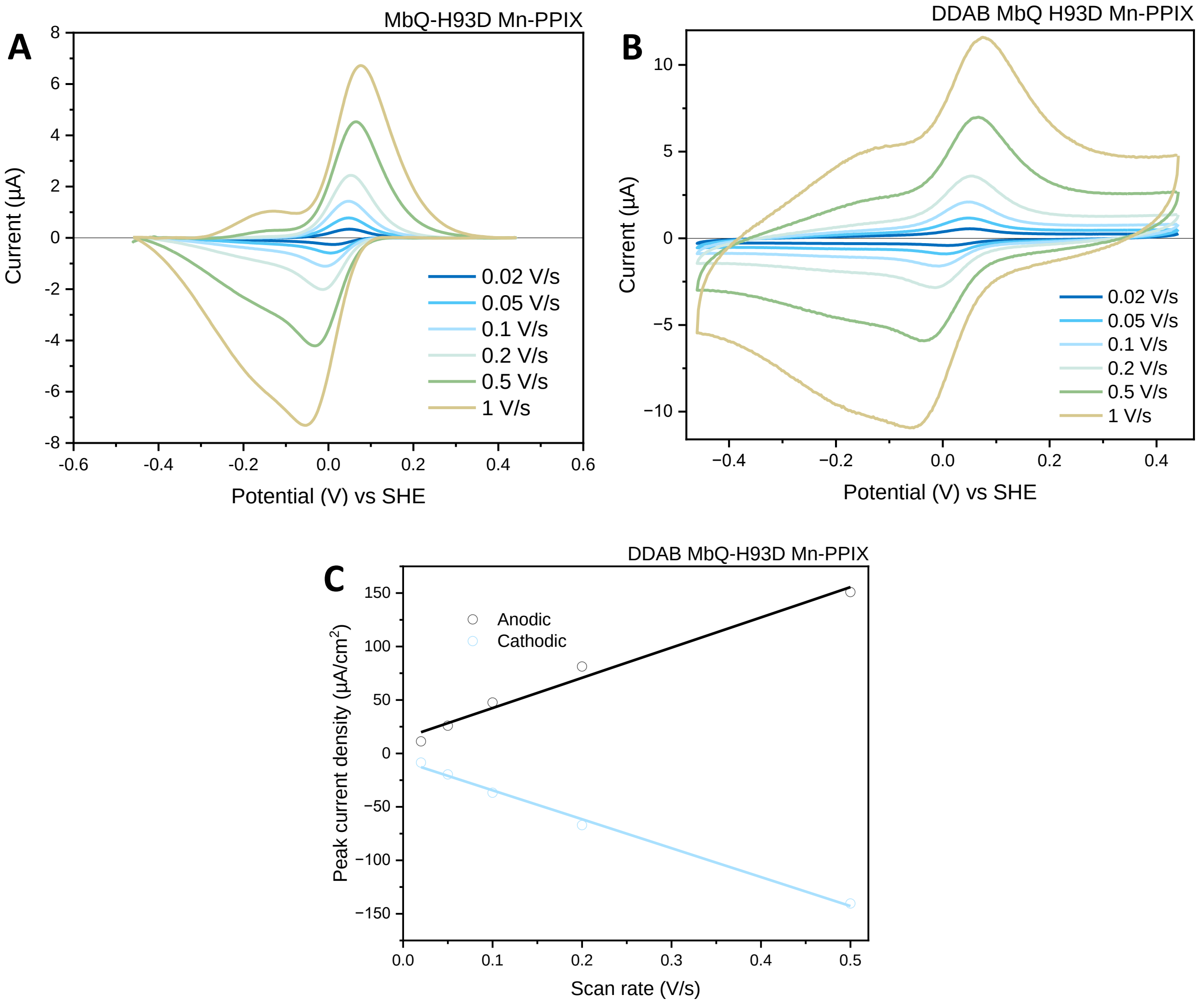

Figure S32. CVs of immobilised MbQ H93D Mn-PPIX in DDAB films on PG electrode surface. A) Baseline subtracted and B) raw varied scan rate CVs are given along with the C) corresponding anodic and cathodic peak current density vs scan rate plot. Measurements were performed under N_2 atmosphere at 25 °C in buffer mix (pH 7) solution using 0.02-1 V/s scan rate.

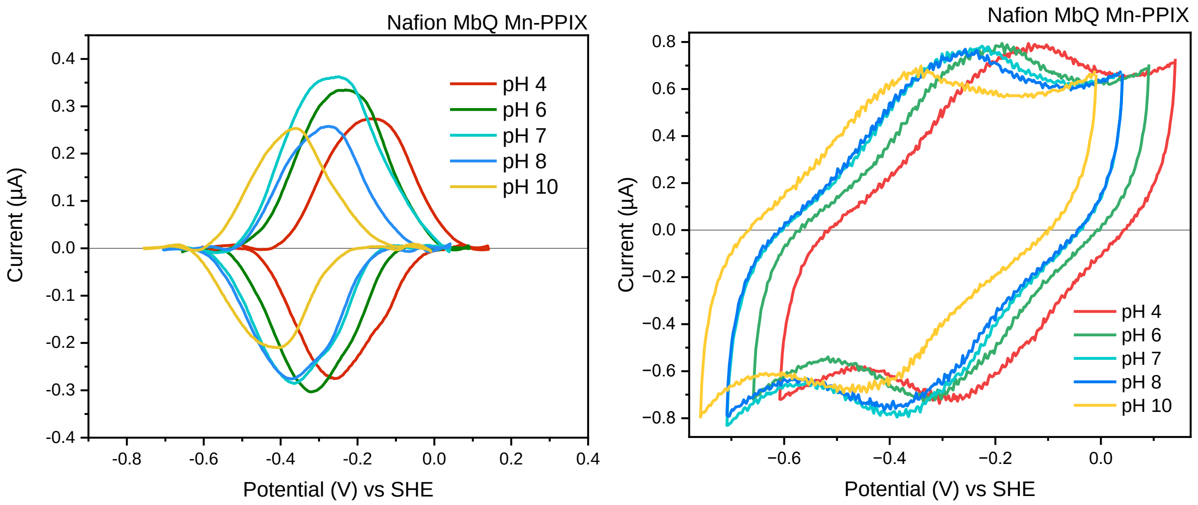

Figure S33. CVs, baseline subtracted (left) and raw (right), of MbQ Mn-PPIX measured adsorbed in Nafion-D521 films on PG electrode surface. Measurements were performed

under N_2 atmosphere at 25 °C in buffer mix solution at either pH 4, 6, 7, 8 or 10 at a scan rate of 0.1 V/s.

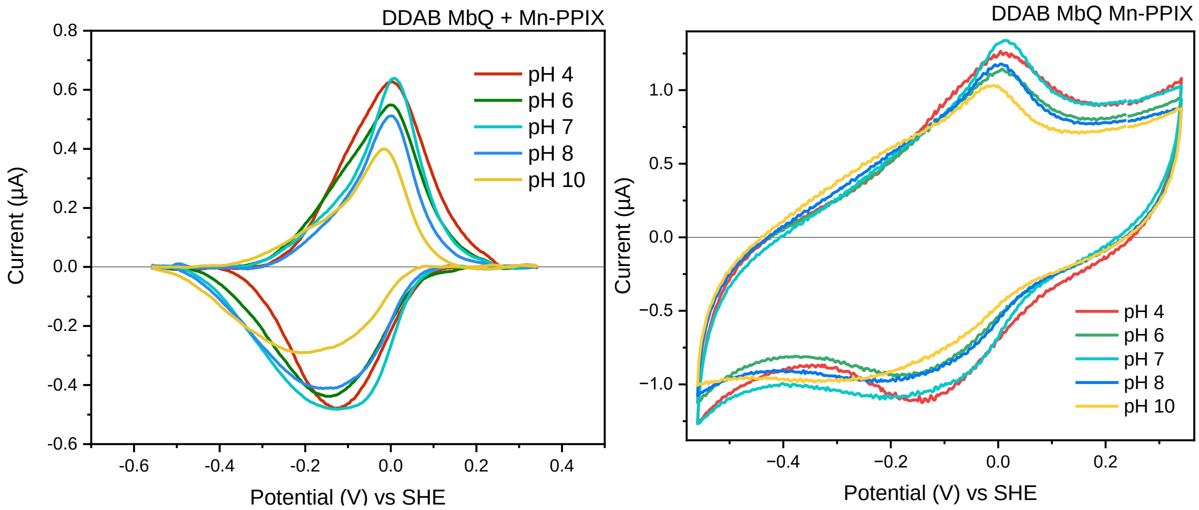

Figure S34. CVs, baseline subtracted (left) and raw (right), of MbQ Mn-PPIX measured adsorbed in DDAB films on PG electrode surface. Measurements were performed under N_2 atmosphere at 25 °C in buffer mix solution at either pH 4, 6, 7, 8 or 10 at a scan rate of 0.1 V/s.

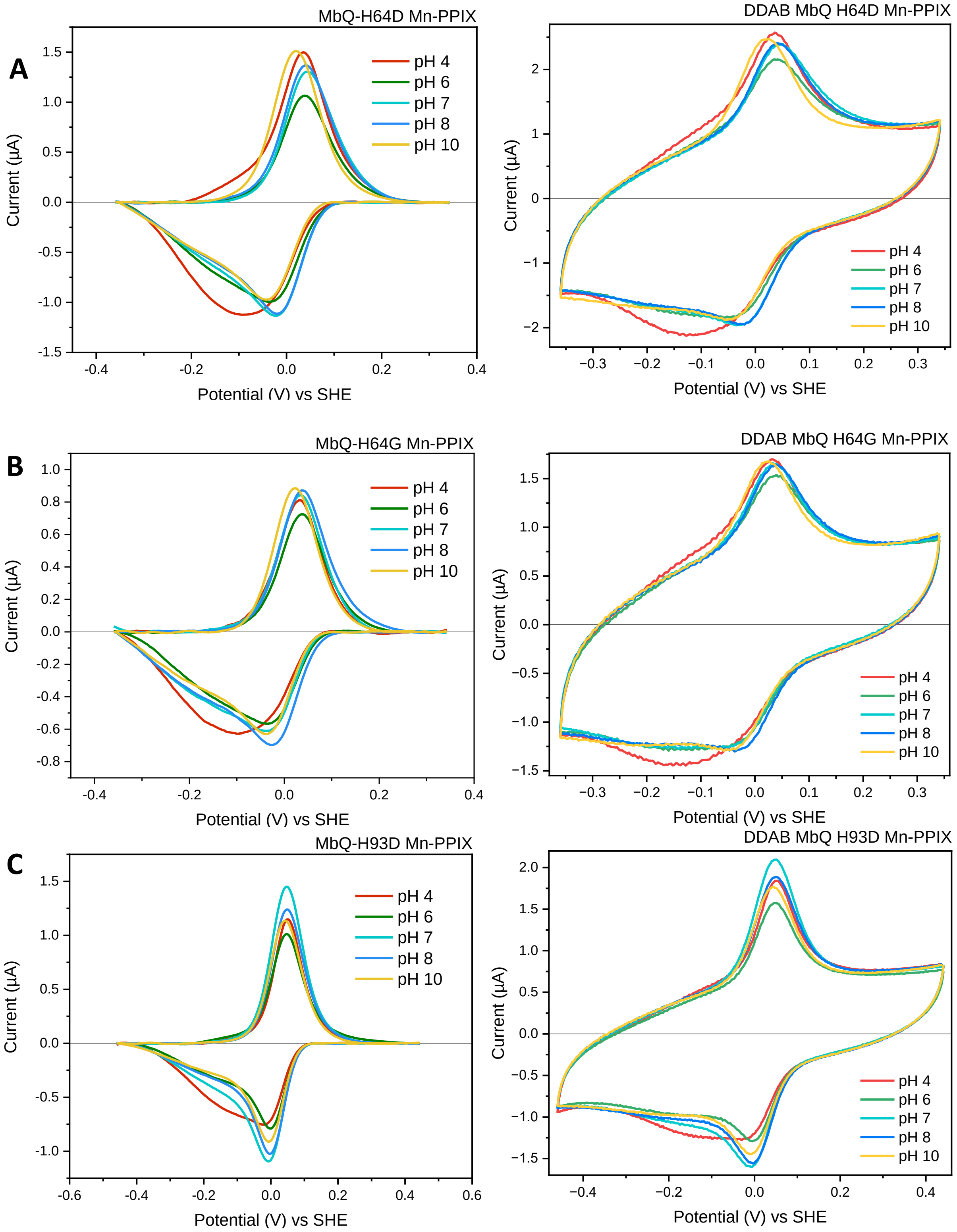

Figure S35. CVs, baseline subtracted (left) and raw (right), of A) MbQ H64D, B) MbQ H64G and C) MbQ H93D bound to Mn-PPIX measured adsorbed in DDAB film on PG electrode surface. Measurements were performed under N_2 atmosphere at 25 °C in buffer mix solution at either pH 4, 6, 7, 8 or 10 at a scan rate of 0.1 V/s.

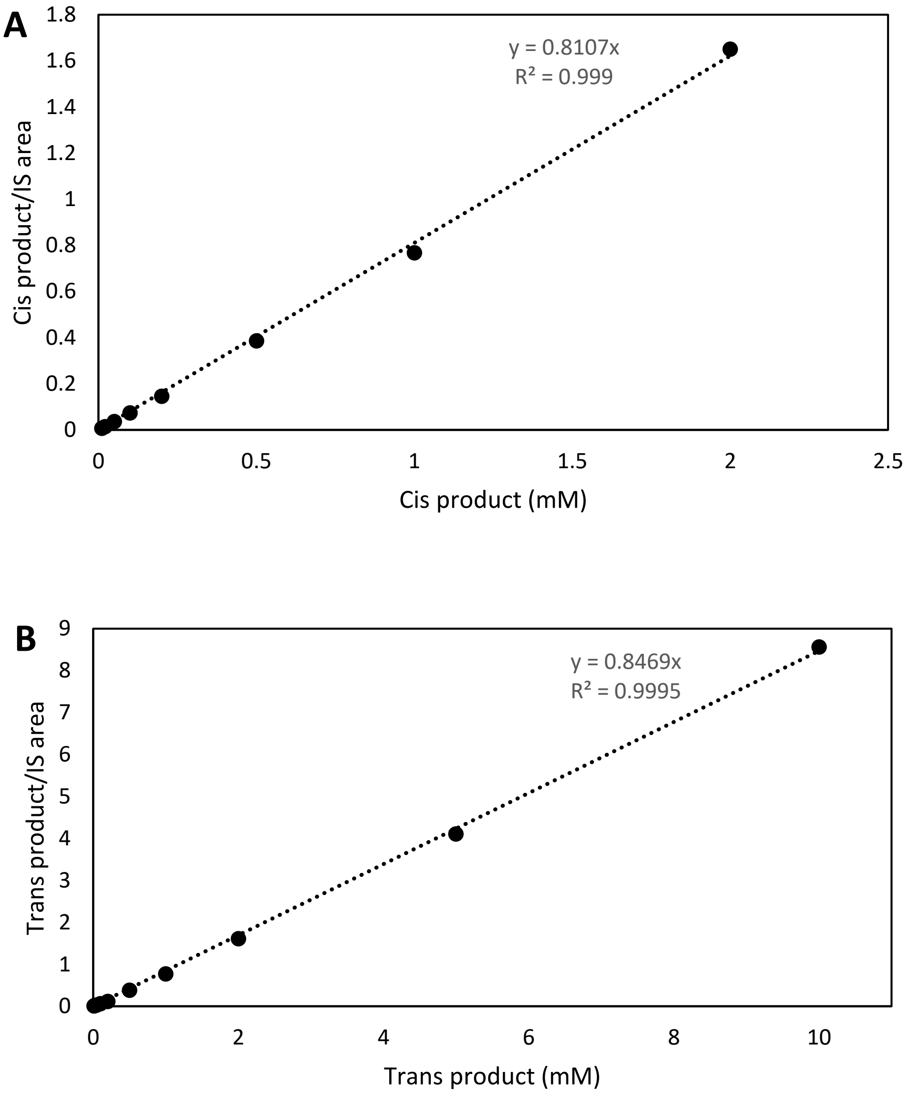

Figure S36. Calibration curves for A) cis and B) trans cyclopropane products using Rxi-5ms column in the GC-FID analysis.

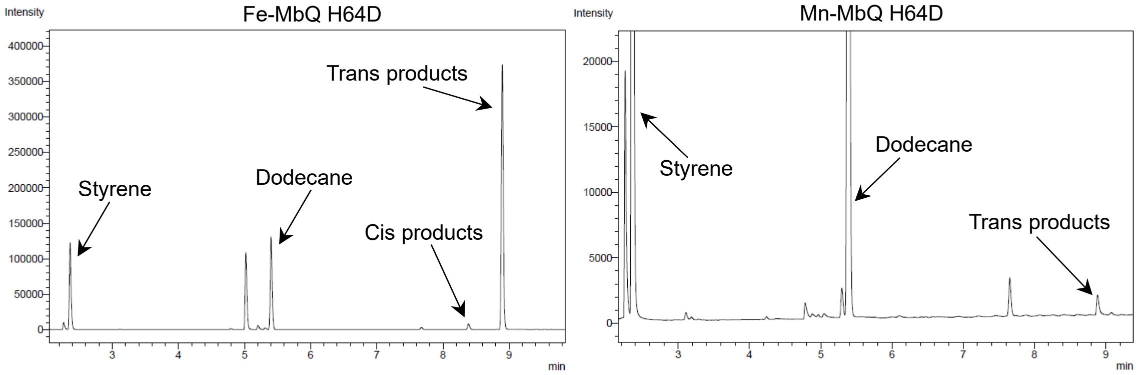

Figure S37. GC traces from the cyclopropanation activity analysis performed using Rtx-5ms column.

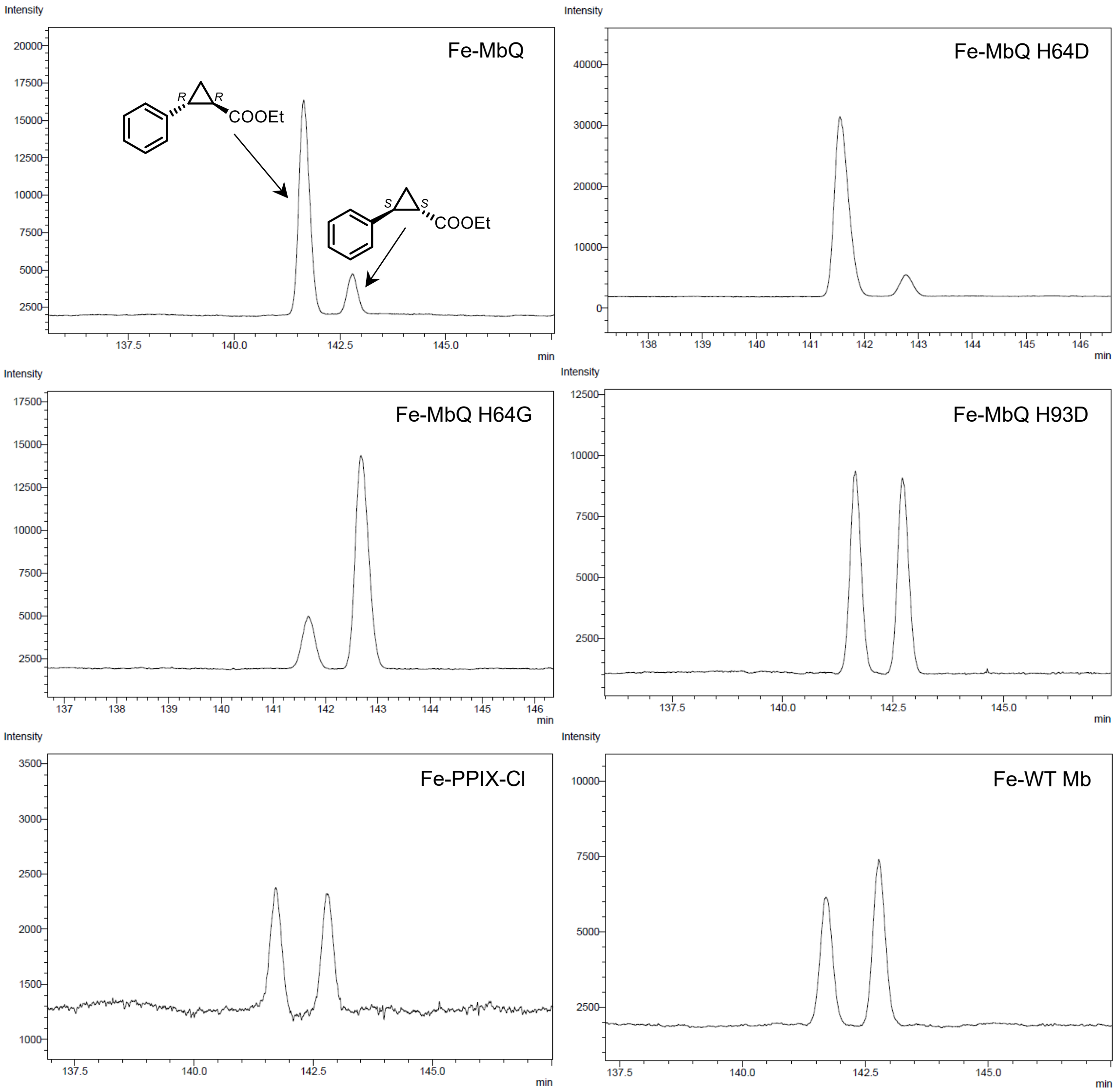

Figure S38. Chiral GC trace analysis of Fe Mb systems. Runs performed using Rt-bDEXm column.

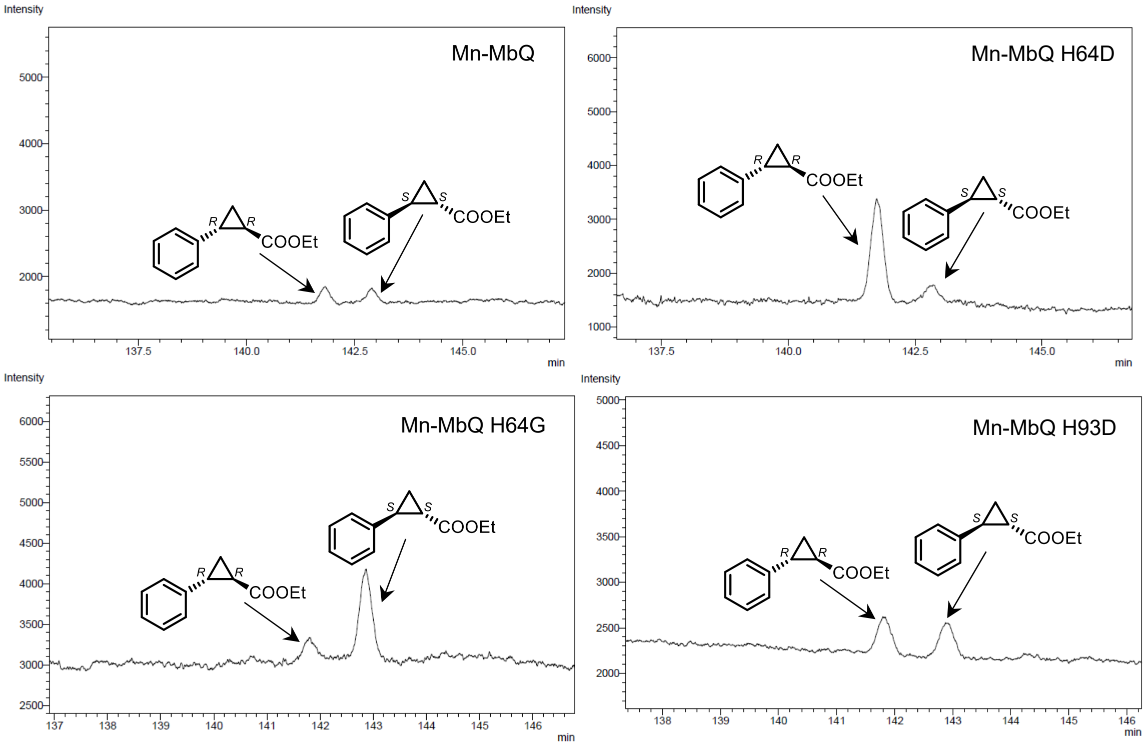

Figure S39. Chiral GC trace analysis of Mn Mb systems. Runs performed using Rt-bDEXm column.

Compound 1 – ethyl 2-cyclophenylpropane-1-carboxylate

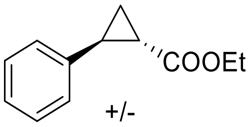

A standard procedure for the preparation of cyclopropanation products was followed as reported in M. Bordeaux, V. Tyagi and R. Fasan, *Angew. Chem. Int. Ed.*, 2015, **54**, 1744-1748. Yield = 50%, *E-isomers*: white solid. ^{1}H NMR ($CDCl_3$, 500 MHz): δ 7.28 (m, 2H), 7.20 (m, 1H), 7.10 (m, 2H), 4.17 (q, *J* = 7.1 Hz, 2H), 2.5 (m, 1H), 1.90 (m, 1H), 1.60 (m, 1H), 1.31 (m, 1H), 1.28 (t, *J* = 7.1 Hz, 3H). ^{13}C NMR ($CDCl_3$, 100 MHz): δ 173.6, 143.9, 128.6, 126.6, 126.3, 60.9, 26.9, 24.3, 17.2, 14.4. MS^+ (m/z): 190 $[M]^+$. *Z-isomers*: yellow oil. ^{1}H NMR ($CDCl_3$, 500 MHz): δ 7.27 (m, 2H), 7.25 (m, 2H), 7.19 (m, 1H), 3.87 (q, *J* =7.1 Hz, 2H), 2.58 (m, 1H), 2.08 (m, 1H), 1.71 (m, 1H), 1.32 (m, 1H), 0.97 (t, *J* = 7.1 Hz, 3H). ^{13}C NMR ($CDCl_3$, 100 MHz): δ 172.2, 137.6, 129.9, 128.0, 126.0, 60.3, 29.6, 21.4, 14.2, 11.3. MS^+ (m/z): 190 $[M]^+$.

^{1}H NMR ($CDCl_3$, 500 MHz) spectrum of *trans* isomer of compound 1.

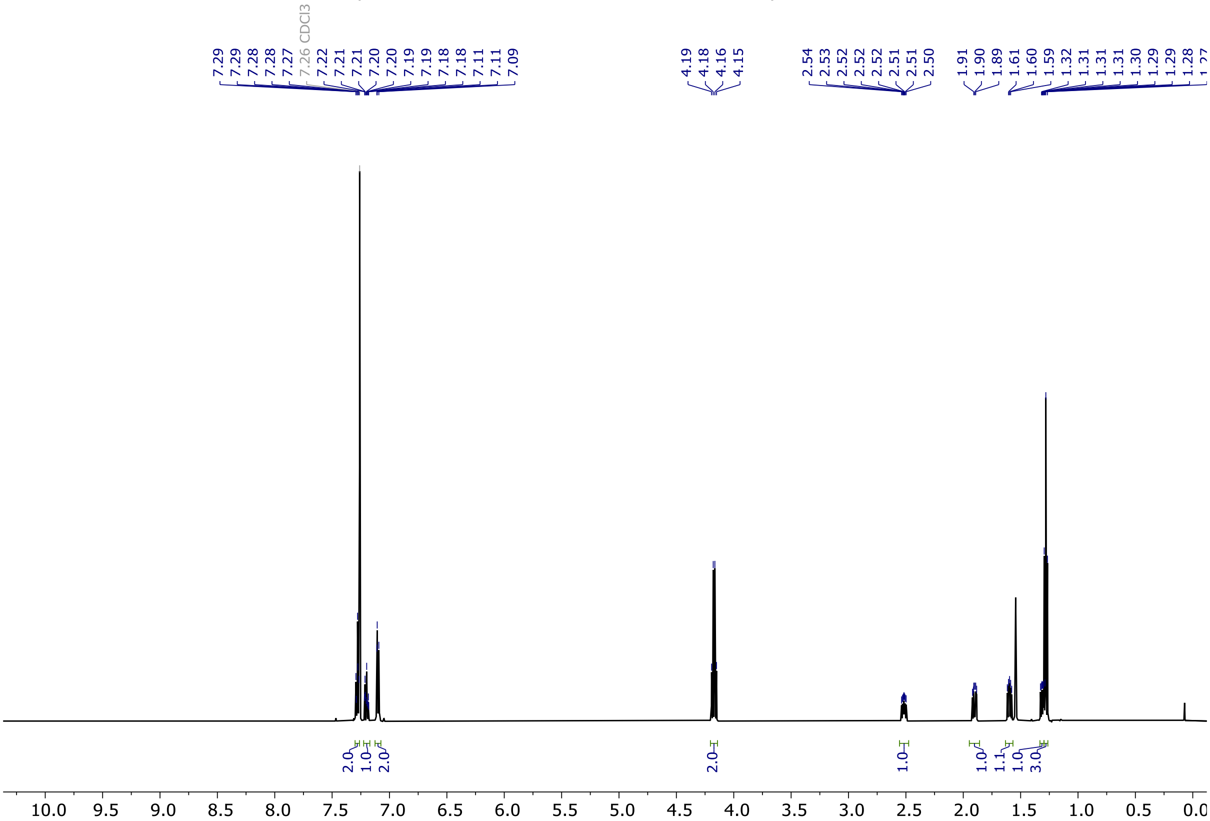

^{13}C NMR ($CDCl_3$, 100 MHz) spectrum of *trans* isomer compound 1.

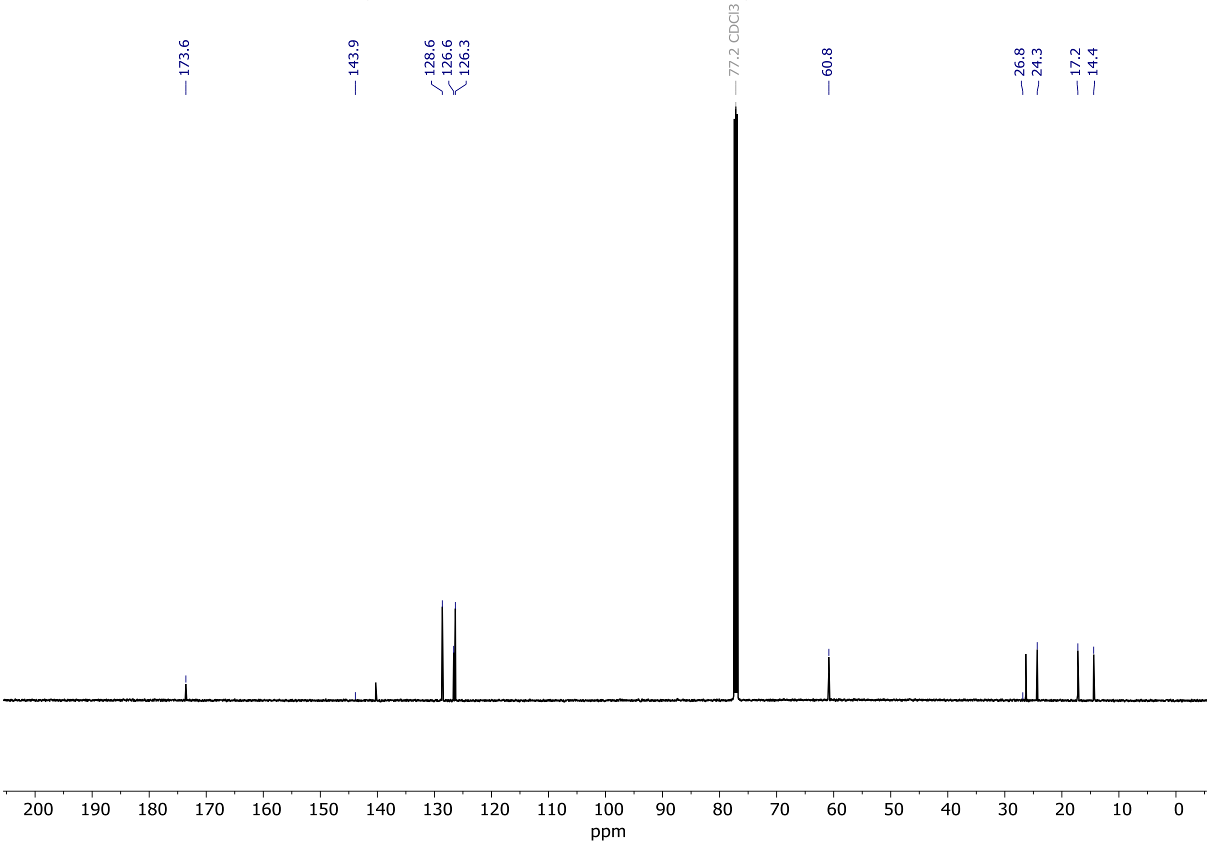

^{1}H NMR ($CDCl_3$, 500 MHz) spectrum of *cis* isomer of compound 1.

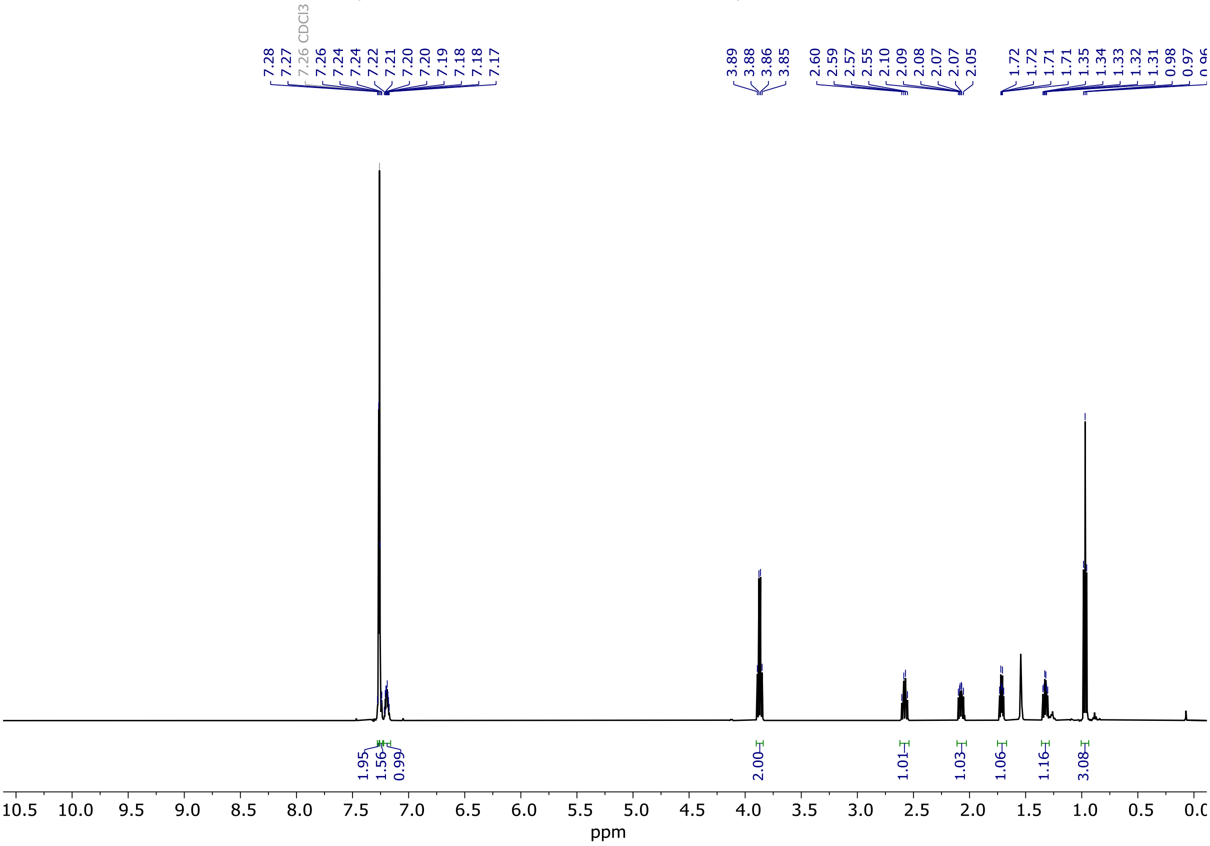

^{13}C NMR ($CDCl_3$, 100 MHz) spectrum of *cis* isomer compound 1.

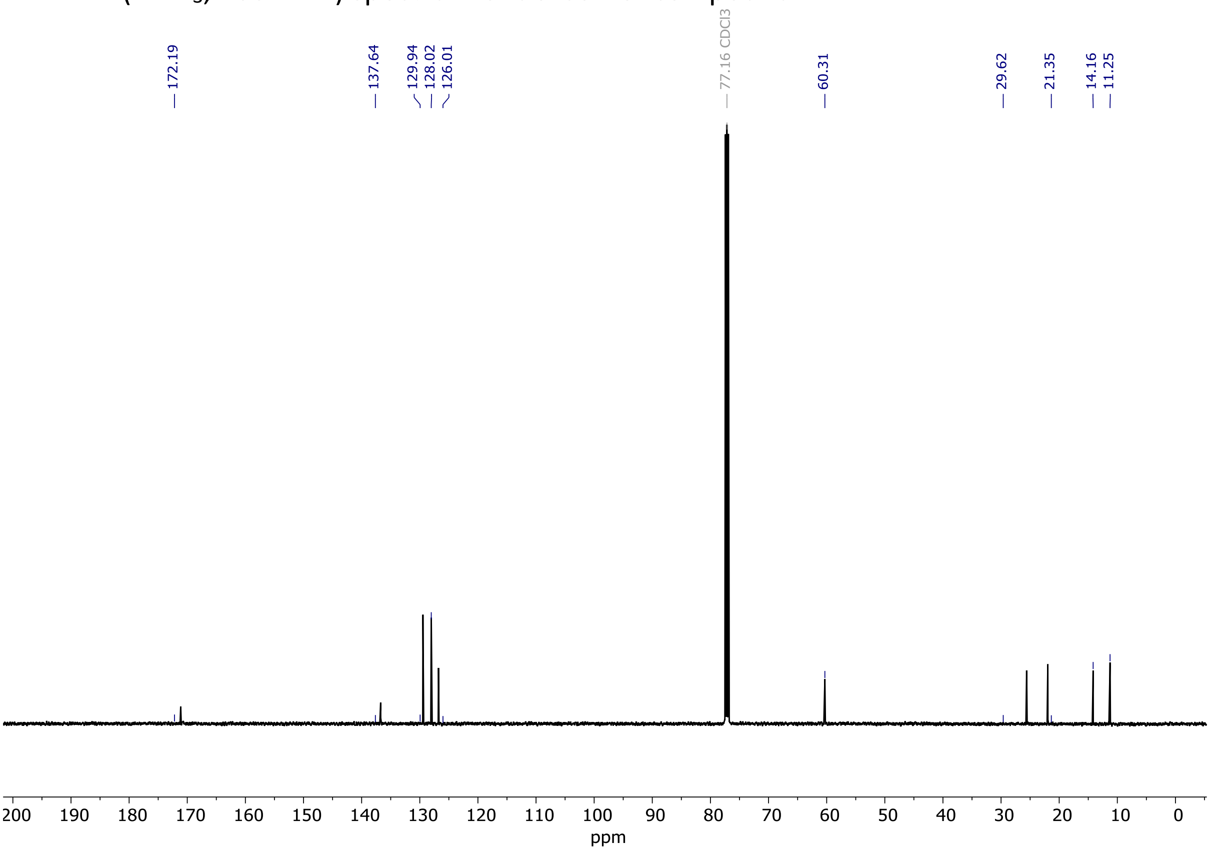

Supplement: DT-OLF-D6DT00266H-s001 [file DT-OLF-D6DT00266H-s001.pdf]
